# Plant-Based Natural Products and Extracts: Potential Source to Develop New Antiviral Drug Candidates

**DOI:** 10.3390/molecules26206197

**Published:** 2021-10-14

**Authors:** Eyana Thomas, Laura E. Stewart, Brien A. Darley, Ashley M. Pham, Isabella Esteban, Siva S. Panda

**Affiliations:** Department of Chemistry & Physics, Augusta University, Augusta, GA 30912, USA; eythomas@augusta.edu (E.T.); lastewart@augusta.edu (L.E.S.); bdarley@augusta.edu (B.A.D.); aspham@augusta.edu (A.M.P.); iestebancastan@augusta.edu (I.E.)

**Keywords:** natural products, extracts, mechanism of action, antiviral activity, drug development

## Abstract

Viral infections are among the most complex medical problems and have been a major threat to the economy and global health. Several epidemics and pandemics have occurred due to viruses, which has led to a significant increase in mortality and morbidity rates. Natural products have always been an inspiration and source for new drug development because of their various uses. Among all-natural sources, plant sources are the most dominant for the discovery of new therapeutic agents due to their chemical and structural diversity. Despite the traditional use and potential source for drug development, natural products have gained little attention from large pharmaceutical industries. Several plant extracts and isolated compounds have been extensively studied and explored for antiviral properties against different strains of viruses. In this review, we have compiled antiviral plant extracts and natural products isolated from plants reported since 2015.

## 1. Introduction

Viruses are small infectious particles ranging from 20 to 300 nm in size and containing nucleic acids, proteins, and lipids [1]. The viruses are simple in their structure, but their interactions with the host are very complex. Viruses have always been a major threat to the economy and global health because of their epidemics and pandemics nature, and they are prone to mutation and resistance to therapy as well [2,3]. There are several examples of viruses that are known to have caused either an epidemic or pandemic in the last twenty years. These include avian influenza A (H5N1) in 1997, paramyxovirus (Nipah virus) in 1999, coronavirus (CoV), known as SARS-CoV in 2002, swine H1N1 influenza A virus in 2009, Middle East Respiratory Syndrome virus (MERS-CoV) in 2012, Ebola outbreak in 2014, and COVID-19, known as SARS-CoV-2, which is seen today (declared as a pandemic by WHO on March 2020). Millions of people have died because of these viruses [4]. As of September 2021, COVID-19 has affected more than 219 million people with 4.55 million deaths worldwide, and the number continues to rise [5]. Currently, there are few preferred antiviral drugs available such as Acyclovir used to treat herpes simplex virus or amantadine used to treat influenza type A, to name a few; unfortunately, of these, none are effective against all types of viruses [6]. Therefore, it is imperative to discover new antiviral drugs. Several FDA-approved drugs (oseltamivir, ritonavir, remdesivir, ribavirin, favipiravir, chloroquine, hydroxychloroquine) are currently being considered for the treatment of COVID-19 (the so-called “drug repurposing” approach) to expedite the process of drug development as well as reduce time and cost [7,8]. Researchers are considering currently available resources (from the synthetic and natural world) for the development of new drugs by molecular modifications of known antiviral scaffolds.

**Figure 1:**
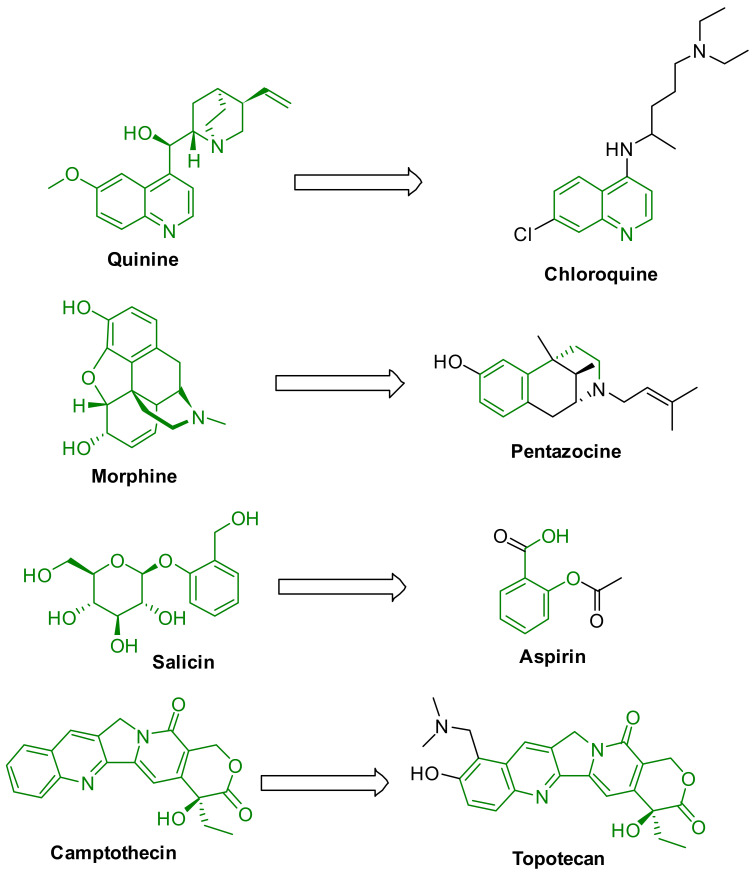
Natural products-inspired synthetic drugs.

Exploring natural products could be an effective strategy to develop new potent antiviral drugs. The world has a long history of using natural products for medical purposes. Among all-natural sources, plant sources are the most dominant for the discovery of new therapeutic agents because of their chemical and structural diversity. Many natural products were identified as potential drugs such as morphine, quinine, paclitaxel, penicillin, digitoxin, lovastatin, berberine, and doxorubicin. In addition, nature in one guise or another has continued to influence the design of small drug-like molecules. Many natural products are used as scaffolds for developing new synthetic drugs such as chloroquine, atorvastatin, captopril, aspirin, and pentazocine (Figure 1) [9]. However, less than 15% of the natural sources have been explored so far, leaving many opportunities in natural product chemistry research. Many review articles have been published on natural products with their diverse uses including antiviral properties [6,10,11,12,13,14,15,16], although few have covered the breadth needed, and an update in the development of drug discovery from the natural sources would provide researchers an effective beginning toward such efforts. We believe that plant-based natural products could play a vital role in developing potential antiviral drug candidates. Recently, several review articles reported on the antiviral properties of plant extracts and isolated compounds [12,17,18]. The previous review articles were focused either on a class of phytochemicals, plant extracts against specific viral strains, or targets [17,19,20,21]. This review aims to provide an update of plant extracts and isolated compounds (secondary metabolites) with structures that show antiviral properties (we have included the EC_50_ or IC_50_ values) since 2015. In addition, we have determined the drug-like properties of the most active isolated antiviral compounds to understand the possible durability as medicinal agents. We believe this review will help the researcher in the design and development of potential antiviral drug candidates.

## 2. Antiviral Activity from Plant Extracts and Secondary Metabolites

Extraction is the initial and most crucial step in the investigation of medicinally important plants. It is crucial to extract the desired chemical components from the plant materials by following the appropriate extraction process for further isolation and characterization, which is an especially challenging task for researchers. Essential precautions must be taken to not lose the activity or the desired component during the process of extraction. Various traditional and modern methods are used to prepare the plant extract from different parts of the plants such as Soxhlet extraction, reflux extraction, sonification, decoction, maceration, pressurized-liquid extraction, solid-phase extraction, microwave-assisted extraction, hydro distillation, and enzyme-assisted extraction [22,23]. Spectroscopic techniques including X-ray studies play an important role in structure determination and confirmation [24].

Extracts are mixtures of secondary metabolites. Most often, the activity of the extracts is not due to a single constituent; instead, the activity may be because of the synergetic effect of two or more active constituents. Diverse classes of compounds are found in plants and their extracts; however, most of the bioactive compounds come from four major classes: alkaloids, glycosides, polyphenols, and terpenes. Several extracts were collected from different parts of various plants and were reported for their antiviral properties against a wide range of strains.

In this section, we have discussed the natural compounds, which were isolated, characterized, and evaluated for their antiviral properties. Their different chemical structures, wide therapeutic use, and the urgent need for new drugs has led to the rising attention of natural products as a source for drug development [2].

### 2.1. Influenza Virus

Influenza is a respiratory virus that affects the nose, throat, and respiratory system [25]. The influenza virus enters the body and attacks healthy cells, typically, epithelial cells. Once the virus enters the healthy cell, it replicates and spreads to infect other healthy cells [25]. The key to stopping the infection from spreading is to not allow the virus to enter the cell and replicate. According to the CDC, 35.5 million people were infected by influenza viruses, and 34,200 people died from the virus during the 2018–2019 influenza season [25]. There are several different classes of influenza: class A, B, C, and D influenza. Classes A, B, and C can infect humans, whereas class D infects cows [26]. Class A influenza is the most common type of influenza and has several subtypes based on its hemagglutinin and neuraminidase surface proteins. The hemagglutinin refers to the HA portion of the subtype, and the neuraminidase refers to the NA portion of the subtype [26]. After each letter, there are corresponding numbers that relate to the different strains. There are 18 different types of HA and 11 different types of NA [26]. A common subtype of influenza is known as A(H1N1), which is the subtype responsible for the 2009 swine flu pandemic. Later, the A(H3N2) variant of A(H1N1) predominated in human infections as a result of inclusion of A(H1N1) variant genetic information in the 2010–2011 seasonal flu vaccine [27]. With influenza rapidly evolving, it is important to be constantly researching and finding other ways to treat this virus [28]. Natural products are one way to find and develop new drugs and tend to be safer and less expensive. Below, we include studies that examine plant extracts’ potency and explore their mechanisms of action.

Brazil, a country known for having the largest biodiversity, has had several lectins isolated and reported for their antiviral properties. Recently, Gondim et al. screened the lectins from the Northeastern Brazilian flora, *Canavalia brasiliensis* (ConBr), *Canavalia maritima* (ConM), *Dioclea lasiocarpa* (DLasiL), and *Dioclea sclerocarpa* (DSclerL) against 18 different viruses. DSclerL and DLasiL exhibited EC_50_ values of 9 nM for HIV-1 and 46 nM for the respiratory syncytial virus (RSV). DLasiL also showed inhibitory property against feline coronavirus at an EC_50_ of 5 nM, and DSclerL, ConBr, and ConM revealed significantly low EC_50_ against influenza A virus strain H3N2 (0.4 nM) and influenza B virus (6 nM) [29].

Wang et al. isolated fractions from the twigs and leaves of *Laggera pterodonta* to evaluate its antiviral properties. Of the fractions, they identified their fraction 14 (Fr 14) as the most active against H3N3 with an IC_50_ of 43.5 μg/mL. It also showed activity against two different H1N1 strains. Through time addition assays, they observed that Fr 14 acts on the early stages of viral replication. Mechanistically, it inhibited the p38/MAPK pathway and further inhibited the COX-2 and NF-kB pathway. Despite these findings, further studies must be done to determine a more detailed mechanism [30].

Yu et al. extracted dried plant leaves from the *Mosla scabra* plant. The extracted compound showed antiviral activity against the influenza A virus (IAV). Data showed that the inhibitory rate of *Mosla scabra* on the lung index of IAV-infected mice treated with the total flavonoids extracted from *Mosla scabra* (MF) (40 mg/kg) was 10.02%; that of IAV-infected mice treated with MF (120 mg/kg) was 33.54%; and that of IAV-infected mice treated with MF (360 mg/kg) was 52.44%. MF was shown to increase the expression of INF-α in the blood and decrease the expression of pro-inflammatory cytokines. This finding suggests that it may not activate the NF-κB and apoptosis pathway. While the mechanism of *Mosla scabra* against IAV is still elusive, these findings suggest a possible avenue for future studies [31].

In addition, there are several plant extracts that were reported for the antiviral properties against various strains of influenza virus, which are listed in Table 1. These extracts’ mechanism of actions were not elucidated and warrant further research.

Of the extracts that are obtained from plant-based material, natural compounds can be isolated, characterized, and explored for their mechanism of actions as anti-influenza agents, such as the following studies.

Inoue et al. investigated the antiviral effects of the extract from the stems and roots of Salacia reticulata on H1N1. The authors observed there is an 80% decrease in the incidence of coughing after oral administration of 0.6 mg/day. The major phytochemical constituents in Salacia reticulata are salacinol, kotalanol, and catechin [45].

**Figure 2:**
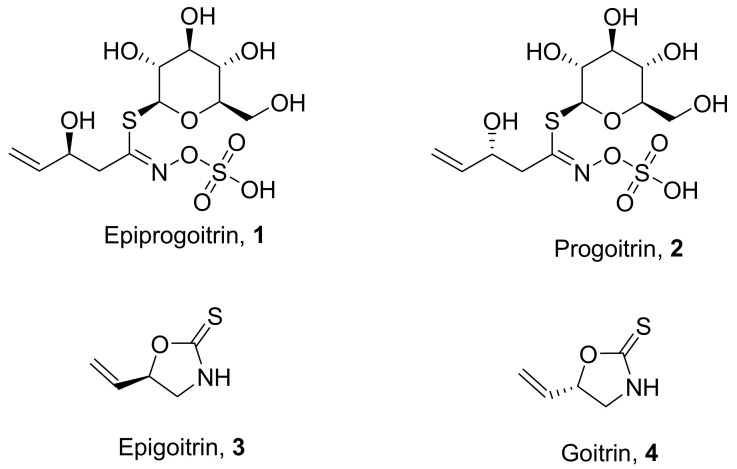
Isolated natural products from *Isatis indigotica*.

Ma et al. isolated four natural products from the sun-dried roots (Isatidis Radix) of the plant *Isatis indigotica* (Figure 2). The isolated compounds showed potential antiviral properties against influenza virus A (H1N1) in the order of progoitrin (**2**) > goitrin (**4**) > epigoitrin (**3**) > epiprogoitrin (**1**). These compounds did not show promising in vitro antiviral activity. However, in vivo studies show activity at a concentration of 5 mg/mL. Hemagglutination (HA) and neuraminidase (NA) inhibition assays were performed to understand the antiviral mechanism, but interestingly, these compounds did not show any inhibition effect, even at higher concentrations [46].

**Figure 3:**
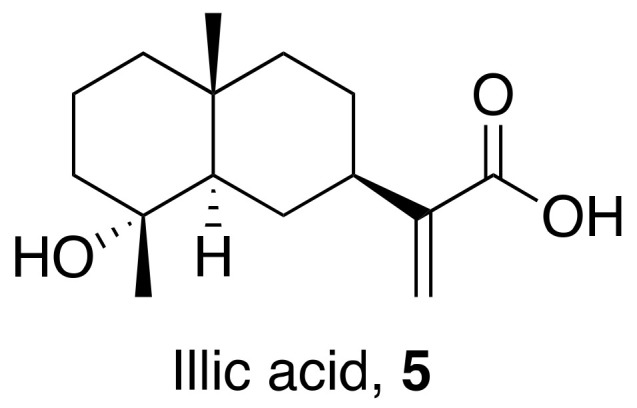
Isolated compound from *Laggera pterodonta*.

Wang et al. isolated and characterized an active fraction from *Laggera pterodonta*. The isolated compound illic acid (**5**) (Figure 3) from the active fraction showed potential antiviral properties against influenza virus A (H1N1 and H3N2) and avian influenza virus (H6N2 and H9N2). In vitro studies show that the active fraction is effective against influenza strains A/PR/8/34 (H1N1), A/Guangzhou/GIRD07/09 (H1N1), and A/Aichi/2/68 (H3N2) with IC_50_ values of 79.4, 43.4, and 75 μg/mL, respectively. However, they are not as effective as the standard Oseltamivir (0.05 µg/mL). Time of addition, bio-plex, and Western blotting assays were performed to understand the antiviral mechanism. The results suggest that the active fraction inhibits the early stage of the virus replication. Western blotting assay results show that the fraction inhibits the p38/MAPK, NF-κB, and COX-2. Lastly, it was shown to increase the expression of cytokines and chemokines [30].

**Figure 4:**
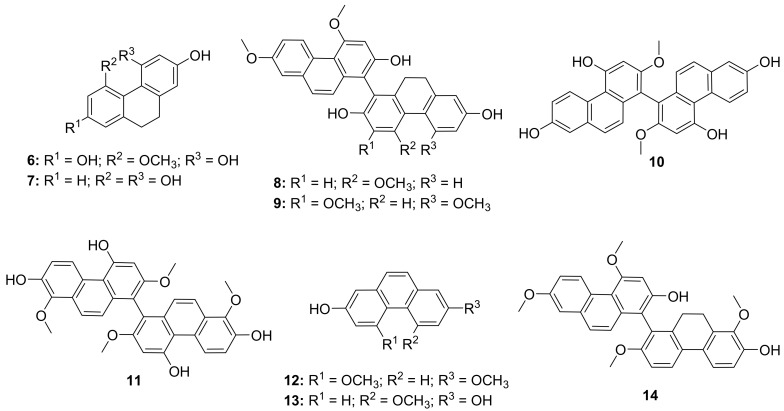
Isolated natural products natural products from *Bletilla striata*.

Shi et al. isolated twelve phenanthrene natural products from *Bletilla striata* (Figure 4). The isolated compounds showed potential antiviral properties against influenza virus A (H3N2) in the order of compound **9** > **6** > **11** = **10** = **7** = **13** > **12** > **14** > **8** and percent inhibition values of 17.2, 20.7, 34.5, 48.8, 75.9, and 79.3% respectively. These compounds did not show promising results as pretreatment. However, in vivo studies show that compounds **8**, **9**, **10**, **11**, **12**, and **14** have strong inhibition in both simultaneous treatment (IC_50_ from 14.6 ± 2.4 to 43.3 ± 5.3 μM) and post-treatment (18.4 ± 3.1 to 42.3 ± 3.9 μM) assays. Hemagglutination (HA) and neuraminidase (NA) inhibition assays were performed to understand the antiviral mechanism. Compounds **6**, **9**, **10**, **11**, **12**, and **13** showed strong inhibition on NA; however, no compound was able to inhibit hemagglutination. Oseltamivir was used as a reference drug for this study (100% inhibition). Lastly, the presence of compounds **8**, **9**, **10**, **11**, **12**, and **14** led to a reduction in transcription of viral matrix protein mRNA [47].

**Figure 5:**
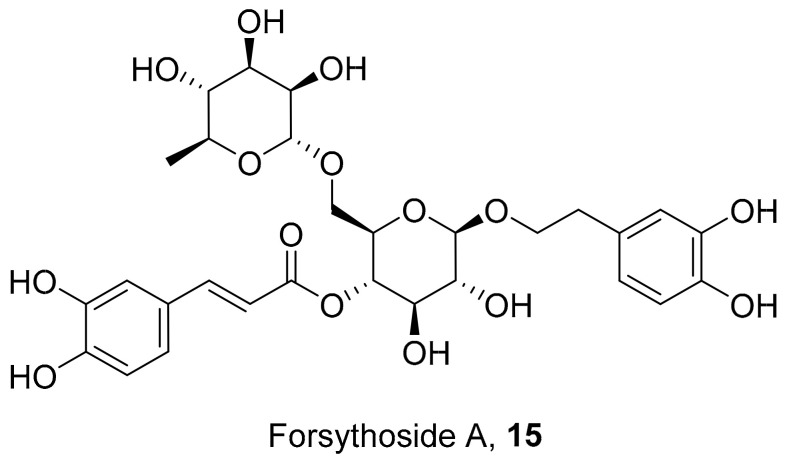
Isolated a compound from *Forsythia suspensa*.

Law et al. isolated a compound from seven medicinal herbs to determine the antiviral activity against influenza (H1N1) viruses (Figure 5). Forsythoside A (**15**) was isolated as the active compound from the fruit of *Forsythia suspensa* and was found to be active against the various influenza subtypes. The treatment of compound **15** led to a slower and abnormal release mechanism of the virus via electron microscopy. Western blotting assay results showed that it reduced M1 protein expression. This may be contributing to the inhibitory effects seen on viral replication. However, the mechanism in which compound **15** leads to the reduced expression still needs further investigation [48].

**Figure 6:**
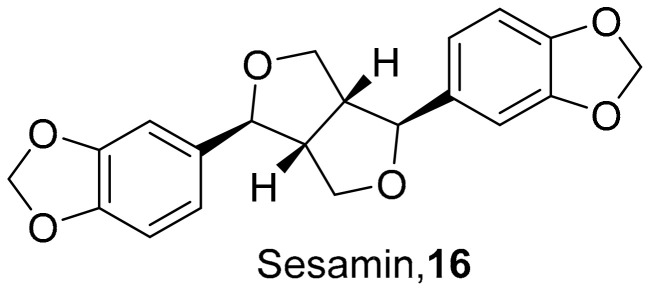
Structure of sesamin (**16**).

Fanhchaksai et al. used combinatorial screening along with computational methods to determine the effects of sesamin (**16**) (Figure 6) on target proteins against influenza H1N1. The computational data from **16** showed promising results that sesamin could be used as an alternative antiviral H1N1 compound. Compound **16** reduced the neuraminidase activity of influenza H1N1. Western blotting revealed that sesamin decreased the expression of pro-inflammatory cytokines via the MAPK pathway at concentrations of 5 μg/mL. However, **16** does exhibit some unwanted side effects, making this sesamin a starting point for future studies to optimize it as a lead [49].

In addition to the above-mentioned compounds, many other compounds isolated from various plants are listed in Table 2 along with their IC_50_/EC_50_ values against influenza strains.

### 2.2. Human Immunodeficiency Virus (HIV)

Human Immunodeficiency Virus type 1 (HIV-1) is a retrovirus that attacks healthy immune cells, thus affecting and eventually destroying the human immune system. More than 33 million deaths have been reported globally since its first major outbreak. However, the outbreaks have been reduced by up to 40% from the initial rates because of prevention strategies and treatment plans [59]. Antiretroviral therapy (ART) has shown success in preventing viral replication and improving the lives of those living with HIV-1 but is not useful in eliminating the virus within the body. As a result of this, many ART drugs are used in combination to treat the infection. This allows for a decrease in the viral load, which may lower the levels of the virus to nearly undetectable amounts [60]. Drug resistance has been documented in all six of the antiretroviral drug classes and as such often requires resistance testing before beginning the therapies. This often requires the use of multiple drugs in combination with ART to have a significant effect on the viral load. The molecular mechanism involved in the reverse transcription process of retroviruses such as HIV-1 makes it likely for errors to occur. Often, these result in mutations and subsequently cause an increased genetic diversity in the HIV-1 virus, ultimately allowing for potential drug resistance [60].

Current HIV-1 treatments have undergone prominent advances over the past three decades since the major outbreak of the virus but are far from being perfect at HIV-1 treatment or prevention. No current treatment can effectively cure the viral infection despite being able to reduce the viral load. The current medications often have side effects and require lifetime administration of the drugs, further burdening the patients. Cessation of medication use can result in a spike in the viral load, increasing the chance of mutations and a need for alterations to patient treatments. Natural products have been researched for suppressing HIV [59]. In its early history, ART drug research did focus on several natural compounds including Calanolides for NNRTI activity, Kuwanon-L from the black mulberry tree *Morus nigra* for anti-reverse transcriptase and anti-integrase activity, Bowman–Birk inhibitor from soybeans used as a protease inhibitor, and many others [61]. As a result of the versatility and the vast number of compounds synthesized from plants, ART small-molecule drug candidates could likely be derived from natural products, allowing for a basic understanding of the pharmacokinetic properties in the anti-HIV-1 activity. Table 3 shows a list of plant species extracts with inhibition data, followed by a recent study that investigates the mechanism actions of an isolated compound that shows potent anti- HIV activity.

**Figure 7:**
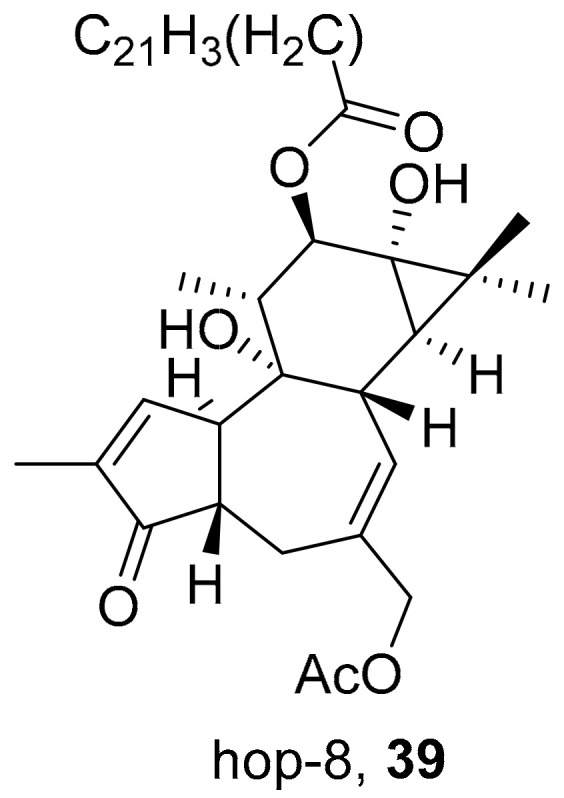
Isolated compound from *Ostodes katharinae*.

Chen et al. isolated a novel phorbol ester, hop-8 (**39**) (Figure 7), from the dried leaves and twigs of *Ostodes katharinae* that shows potent antiviral activity against wild-type HIV-1 and HIV-2 and drug-resistant strains in peripheral blood mononuclear cells (PBMCs). The cytotoxicity assay of Hop-8 shows EC_50_ values ranging from 0.396 to 6.915 μM, which are better than the standard, Prostratin. To determine the mode of action for hop-8, Western blotting and cell transfection techniques were used. One of Hop-8′s mechanisms to resist infection by HIV verified in this study involves stimulating A3G expression, which prevents Vif-mediated degradation. This discovery may be considered as a potent strategy for therapeutic development in the future [68]. Other isolated natural compounds have been found to show significant anti-HIV properties (Table 4) and may also be considered to serve as therapeutic agents.

### 2.3. Arthropod-Borne Flaviviruses

Dengue virus (DENV), West Nile virus (WNV), and Chikungunya virus (CHIKV) are examples of mosquito-borne RNA viruses apart of the Flaviviridae family that cause flu-like symptoms transmitted by the *Ae albopictus* mosquito. Of these viruses, DENV is the most prevalent mosquito-borne virus affecting up to 400 million people each year globally according to the CDC [82]. There is an available vaccine; however, the vaccine may lead to a higher risk of developing severe DENV symptoms for individuals not previously infected with the virus and is only effective in the age group 9–45 years old [83]. DENV has four different serotypes (DENV 1–4). Currently, there are no anti-DENV therapeutics that have been approved by the FDA; however, there are several in the clinical trial phase. The most targeted proteins when developing these anti-DENV drugs include but are not limited to the envelope protein, methyltransferases, and genes important for coding nonstructural (NS) proteins, such as RNA polymerases, protease, and helicase, to name a few [80]. NS2B, NS3, and NS5 are examples of NS protein targets that may be important in developing these therapeutics [84]. Given the lack of availability of therapeutics, natural extracts and compounds may provide a helpful avenue to discover lead compounds or natural treatments. For example, Angelina et al. found that the ethanol extract from the leaves *Cassia Alata* effectively inhibited DENV serotype 2 infection in every step of the virus’s replication cycle [85].

WNV is the leading cause of mosquito-transmitted disease in the continental United States [86]. There are no vaccines for prevention or therapeutics against WNV available. Therefore, there is an urgent need for research for the development of therapeutics against this virus. Potential targets include but are not limited to the envelope protein, NS proteins 3 and 5 [87]. Some natural product extracts are active against WNV, and once the active compounds are structurally elucidated, they may serve potent anti-WNV therapeutics [88].

CHIKV viral infection is mainly seen in countries in the Eastern hemisphere such as Africa, Asia, Europe, and other tropical and subtropical islands [89]. Currently, there are no vaccines or therapeutics available against CHIKV. Similar to DENV, there are current potential targets that include but are not limited to NS proteins 1 and 2, CHIKV capsid, and proteins important to fusion. However, there are many proteins involved in the CHIKV replication cycle that have no structural information. There has been extensive screening for activating anti-CHIKV natural products; while most only show moderate activity, they may serve as lead compounds for developing helpful therapeutics [90]. There is an obvious need for discovery in preventative and curing treatments for these and other viruses, which is a need that may be satisfied through natural sources. Extract isolates in the below studies show inhibitory effects that were further studied to decipher the mechanism in which the extracts manifested its inhibitory effects.

Panya et al. extracted the bioactive peptides of 33 Thai medicinal plants via digestion to evaluate the antiviral activity against DENV. Of these plants, *Thunbergia laurifolia Lindl* and *Acacia catechu* peptide extracts showed potent antiviral activity against DENV. Foci-forming unit assay results showed that both peptide extracts have inhibitory potential with an IC_50_ value of 0.18 and 1.54 μg/mL for *Acacia catechu* and *Thunbergia laurifolia Lindl*, respectively. *Acacia catechu* peptide extract was further studied to determine the active peptide sequence and mechanism of action. The identified peptide inhibitors in the extract of *Acacia catechu* were also found to be effective against all four DENV serotypes. The results of the time of addition assay suggest that the *Acacia catechu* peptide extract inhibits DENV2 in the early stages of infection; however, the mechanism in which the peptides inhibit the early step of infection is still unknown [91].

Leite et al. sought to find the anti-DENV-2 activity of extracts from the leaves of *Cissampelos sympodialish*. Using MTT assays and infection of Huh-7 cells with DENV-2, researchers found that the leaf hydroalcoholic extract (AFL) showed significant inhibitory effects at 10 μg/mL. AFL did not decrease the expression of DENV but rather the expression of cytokines important in its infections. Then, 72 h after infection with the virus showed there was a decrease in the production of migration inhibitory factor (MIF) and TNF-α, both of which are important in proliferating the effects of the DENV infection. Further isolation and elucidation of two AFL alkaloids were found to have no activity, therefore eliminating them as the components responsible for AFL’s activity. Further research must be done to isolate the active components of this fraction to give a clear mechanism of action for AFL [92]. A list of plant species with IC_50_/EC_50_ data is summarized in Table 5.

**Figure 8:**
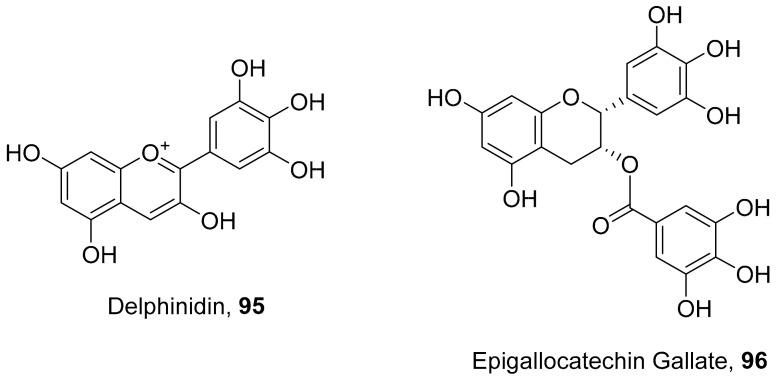
Structure of polyphenolic compounds delphinidin (**95**) and epigallocatechin gallate (**96**).

Vazquez-Calvo et al. used many polyphenols found in green tea and wine to analyze their effects against the West Nile virus (WNV), Zika virus (ZIKV), and Dengue virus (DENV). Cell viability assays, Quantitative-PCR, and LysoSensor assays were done to determine the effect of these polyphenols. Of the different polyphenols tested, delphinidin (**95**) and epigallocatechin gallate (**96**) (Figure 8) showed the most inhibition against WNV at 10 μM. To determine the action on the viral particle, the two polyphenols were added at different times of infection. Compounds **95** and **96** were found to affect the early stages of infection by a suggested virucidal effect. The LysoSensor assay supports **95** and **96** effects via virucidal effect rather than a pH-dependent fusion. The investigation supports that the mechanism of action is due to a direct effect on the viral particle rather than a pH-dependent mechanism. Compounds **95** and **96** also show inhibitory activity against ZIKV and DENV [98].

Many secondary metabolites have been reported to have antiviral properties against arthropod-borne flaviviruses. In Table 6, we have mentioned the compounds that exhibited viral inhibition with inhibitory activity with IC_50_ or EC_50_ dose.

### 2.4. Herpes Simplex Virus

Herpes simplex virus (HSV), commonly known as herpes, is typically found in two different strands: herpes simplex virus type 1 (HSV-1) and herpes simplex virus type 2 (HSV-2). HSV-1 is a highly contagious infection typically transmitted by oral contact but can also be contracted via oral–genital contact [104,105]. HSV-1, based on 2016 statistics, affected approximately 3.7 billion globally in individuals under 50 years old [105]. Typically, individuals infected with this strain are asymptomatic, so much so that infected individuals are unaware that they are carriers. Often, infected patients present with painful ulceration or blisters on or around their mouth [105].

HSV-2 is considered a sexually transmitted infection, and it is almost exclusively transmitted through genital–genital contact [100]. In 2016, HSV-2 affected approximately 419 million people globally within the age group of 15–49 [101]. Individuals with this strain also experience similar ulceration and blisters by their genitals. Newly infected patients may also experience mild cold symptoms, including fever, body aches, and swollen lymph nodes. Transmission is highest in HSV-2 patients when sores are present; however, they can still pass the virus when they are asymptomatic [105]. HSV-2 has been shown to increase the likelihood of contracting HIV by weakening the skin and mucous membrane responsible for protection [106].

According to the CDC, there is currently no cure for HSV [107], but many medications are used to lessen the severity of an outbreak and the frequency at which the virus enters its lytic cycle. Acyclovir, famciclovir, and valacyclovir are examples of medications that can help patients control the symptoms of an outbreak [105]. In 1988, Dr. Gertrude Elion obtained the Nobel Prize for the discovery of acyclovir, which is one of the first-line drugs currently used for the treatment of HSV infections. Studies also suggest HSV reactivation in the brain can lead to Alzheimer’s disease (AD) [108]. Despite their efficiency in symptomatic patients, they do not cure symptoms or entirely prevent HSV transmission. Further research must be done to lessen symptoms for those infected and develop a vaccine to prevent the transmission.

Tannins are an important class of compounds isolated from plants and possess various biological properties, including antiviral properties. Vilhelmova-Ilieva et al. study the antiviral properties of ellagitannins against HSV over the last decade [109]. They also investigated the effect of ellagitannins on acyclovir (ACV)-resistant herpes. The ellagitannin(s)–ACV combination applied against ACV-resistant HSV-1 produced a much stronger synergistic effect compared to the effect observed against ACV-resistant HSV-2 [110].

Bisignano et al. examined the anti-HSV-1 activity of the methanolic extract of *Prunus dulcis*, specifically from the almond skins. Using plaque-forming assays, Western blotting, and other techniques, they were able to demonstrate the extract’s activity in blocking the replication of HSV-1 particles. It was also found that the extract was able to prevent the absorption of the virus in Vero cells. This effect was observed after 1 h post-infection when cells were treated with 0.4 mg/mL of the extract. Further studies must be done to determine the mechanism by which this inhibitory effect takes place [111]. Table 7 summarizes other plant species’ extracts that show inhibitory effects against HSV.

**Figure 9:**
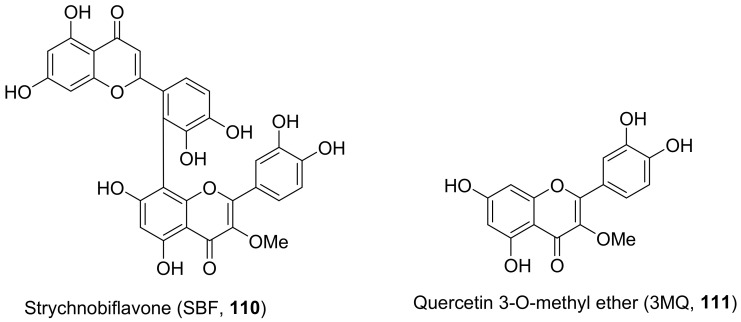
Isolated compounds from *Strychnos pseudoquina*.

The mechanisms of action of standardized ethyl acetate extract from the stem bark of *Strychnos pseudoquina* (SEAE) and isolated compound strychnobiflavone (SBF, **110**) were shown to affect the early stages of viral infection accompanied by reduced HSV-1 protein expression. Both flavonoids (Figure 9) elicited a concentration-dependent inhibition of monocyte chemoattractant protein-1(MCP-1), whereas (3MQ, **111**) reduced the chemokine release more significantly than SBF. Conversely, both compounds stimulated the production of the cytokines TNF-a and IL-1 in LPS-stimulated cells. It can be concluded that SEAE and SBF interfered with various steps of the HSV replication cycle, mainly adsorption, post-adsorption, and penetration, as well as with band c viral proteins expression. Incidentally, the direct inactivation of viral particles was observed. The results are significant as they suggest that the compounds present anti-HSV and anti-inflammatory activities [121].

In Table 8, we have mentioned the compounds that exhibited inhibitory activity on viral inhibition against HSV with the IC_50_ or EC_50_ dose.

### 2.5. Hepatitis Virus

Hepatitis is a viral infection of the liver that causes inflammation, leading to either short-term or long-term damage to the organ’s structure and function and ultimately to the individual. The most prevalent hepatitis strains in the world are Hepatitis A (HAV), Hepatitis B (HBV), and Hepatitis C virus (HCV) [131]. While all three classes of the virus are similar in their responses to the body, they differ in symptoms and treatments.

Of the three, Hepatitis A is considered to be the most contagious. The virus is easily ingested through contaminated food or proximity of infected individuals. However, some are unaware of their infection status unless they are tested, as the symptoms are minor and common to other illnesses. Although Hepatitis A is easily spread, the virus’s short-term effects, minor symptoms, and easy prevention through vaccinations categorize it as the least dangerous [132].

Hepatitis B is also vaccine-preventable; however, HBV is spread internally through bodily fluids from sexual intercourse or injections with contaminated needles. Symptoms of infection may or may not show, and effects can be short-term or long-term. Tenofovir and entecavir are examples of antivirals used to suppress viral replication and other complications associated with an active outbreak. Unfortunately, there are no cures for this viral infection [133].

Similar to Hepatitis B, HCV individuals can be asymptomatic. HCV also has a slight potential to have short-term effects but mainly leads to life-threatening issues [133]. Many people infected with HBV and HCV do not realize they have the virus and spread it mainly through blood from injections or even sexually through open wounds, making them more dangerous than HAV.

Hepatitis is a global issue, and over 320 million people worldwide are affected with just HBV and HCV alone. These statistics do not include consideration of those living unknowingly with the infection [128]. Not only are individuals living with these illnesses, but hepatitis contributes to a large portion of liver cancer cases, leading to close to 2 million deaths per year just from liver cancer [133]. While current therapies and preventions exist against hepatitis, they lead to undesirable and painful side effects and sometimes ineffective treatments. Therefore, it is essential to continue studying hepatitis viruses to develop and improve vaccinations of this disease to make preventative therapies more effective. Traditionally, plant sources are dependable resources for antivirals for hepatitis infections, as we do not have better treatment plans. Several plant extracts were reported and summarized in Table 9 for their antiviral properties against hepatitis A, B, and C.

Effective plant extracts against hepatitis infection were further investigated to identify the active component/molecules responsible for the antiviral properties. The list of isolated compounds was summarized in Table 10 with their IC_50_, EC_50_, and/or CC_50_ values.

In addition to the above discussed natural products against viruses that affect diverse demographics, there are many other natural products reported against viruses such as HCoV, PRRSV, MNV-1, CV-B, HR3V, RSV, etc., that target more specific demographics. The variation in antiviral activity reflects that plant sources are the treasure of lead compounds for the development of antiviral agents.

Cheng et al. evaluated the antiviral activity of extracts from leaves and twigs of *Houttuynia cordata* against MNV-1. There were three extracts obtained: the aqueous extract (HWE), the purified polysaccharide from the aqueous extract (HP), and the ethanolic extract (HEE). The plaque assay results showed that HWE had the most potent antiviral activity with the highest selectivity index of 16.14 with HP having a lesser effect and HEE having the lowest antiviral activity. Previous literature identified that the aqueous extract exhibited great antiviral activity and therefore, HP was further studied to determine its mechanism. Structural analysis suggests that HP may be a pectin-like acidic polysaccharide with a 1,4-linked Galp core. Using time- and dosage-dependent studies, HP was found to reduce the residual infectivity after 10 min of incubation. The mechanism was further studied, and HP was found to be responsible for deforming and inflating viral particles per the results of decimal reduction time and transmission electron microscopic studies. Therefore, HP’s antiviral mode of action inhibits viral penetration in target cells [160].

Different parts of the plant *Nuphar lutea* L., also known as yellow water lily, are used to treat various diseases such as inflammation and pathogen-related diseases. Winer et al. reported the effect of methanolic extract of *Nuphar lutea* leaves on the measles virus (MV). The antiviral property against MV was quantified by using qRT-PCR and the IC_50_ value was determined (0.3 μg/mL). The authors also claim the inhibitory activity of the methanolic extract against Respiratory Syncytial Virus (RSV) [161].

**Figure 10:**
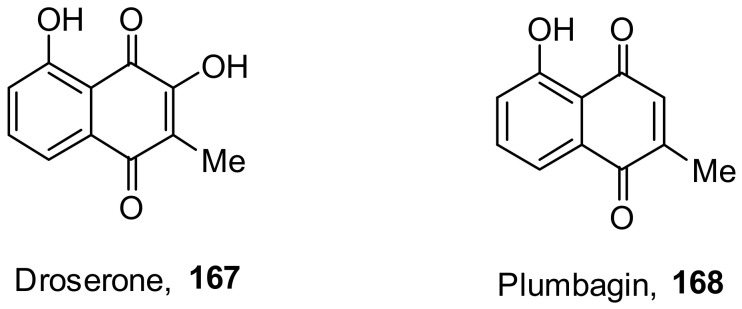
Isolated compounds from *Triphyophyllum peltatum*.

Lieberherr et al. isolated two natural compounds, droserone (**167**) and plumbagin (**168**), from *Triphyophyllum peltatum* (Figure 10). Other structurally similar naphthoquinones were synthesized to determine their antiviral potential against Measle Virus (MV). Infection inhibition and cell viability assays were performed on the compounds. The results showed that droserone had inhibitory activity against MV. Further analysis using the addition of droserone at a different time of the MV cycle found that it must be present during the early stages of infection to inhibit MV. To verify if droserone was acting on the virus or the cells, the cells were preincubated with the compound and then washed, and the absence of droserone showed no significant inhibition. The results suggest that the inhibition of MV is due to interaction with the viral particle. The results from the plaque assay showed that droserone may have interactions with receptor recognition and/or membrane fusion induction processes [162].

Porcine reproductive and respiratory syndrome virus (PRRSV) is endemic in most pig-producing countries. The infections because of this virus affect enormous economic losses to the swine industry. Arjin et al. reported the strong inhibition (IC_50_: 625–1250 μg/mL) of PRRSV replication by the ethanolic extract of the whole plant of *Caesalpinia sappan* and *Tiliacora triandra* [163].

Thabti et al. investigated the water and water–alcohol plant extracts of leaves and stem bark of three different species of mulberry—*Morus alba* var. *alba*, *Morus alba* var. *rosa*, and *Morus rubra*. The authors observed that the leaves’ water–alcohol extracts exhibited maximum antiviral activity on human coronavirus (67–100% inhibition), while stem bark and leaves’ water and water–alcohol extracts were the most effective on picornaviruses (3–15% inhibition) [164].

Human rotavirus (HRoV) is known as the leading cause of severe gastroenteritis in infants and children under the age of five years. Unfortunately, there is no specific antiviral drug for this virus. Civra et al. reported that the methanolic extract of *Rindera lanata* (Boraginaceae) showed the most favorable selectivity index with EC_50_: 25.5 μg/mL. The authors also confirm that the methanolic extract was inactive or barely active against other RNA viruses, namely human rhinovirus and respiratory syncytial virus (RSV) [165].

Coxsackievirus B (CV-B) is a small nonenveloped single-stranded and common enterovirus that produces central nervous system disease as well as various systemic inflammatory diseases. Snene et al. determined the antiviral property of ethyl acetate and methanolic maceration extraction of aerial parts of the plant *Daucus virgatus (Poir.) Maire* by the plaque reduction assay. The authors claim that ethyl acetate and methanol extracts exhibited significant inhibitory effects against CV-B4 virus with IC_50_ values of 98.16 and 60.08 μg/mL, respectively. The cytotoxicity study of the crude extracts on the HEp-2cell line indicates moderate toxicity [166].

**Figure 11:**
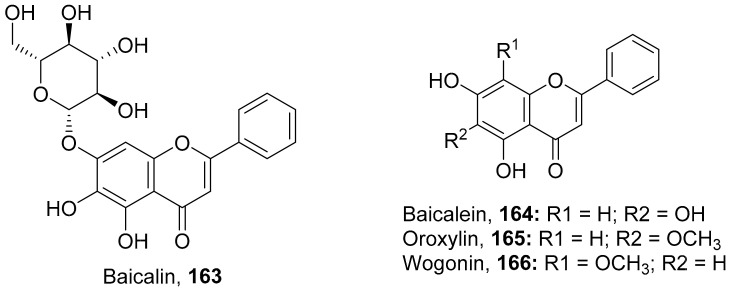
Isolated compounds from *Scutellaria baicalensis*.

It was hypothesized that the compounds baicalin (**163**), baicalein (**164**), oroxylin A (**165**), and wogonin (**166**) (Figure 11) of *Scutellaria baicalensis* extracts (SBE) were capable of interacting with one another to play an effective role against coxsackievirus group B type 3 (CVB3) via various signaling pathways, although this needs further investigation. The data suggest that there was an inhibitory effect on CVB3 viral-induced myocarditis accompanied by a downregulation of the AKT and p38 expressions in viral-infected primary myocardial cells and a viral myocarditis animal model. The results demonstrated that SBE has anti-CVB3 properties both in vitro and in vivo, which are capable of repairing tissue injury and prolong survival in mice with viral myocarditis. However, the exact compounds and the molecular mechanisms by which SBE mediates these antiviral effects against CVB3 remain to be elucidated. From the study, it is heavily indicated that SBE possesses potent antiviral activity with a significant effect on the survival and pathological changes in CVB3-induced myocarditis [167].

**Figure 12:**
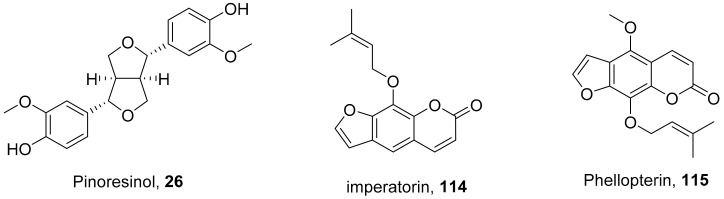
Isolated compounds from *Curcuma aeruginosa* and *Angelica archangelica*.

The compound (pinoresinol, **26**) isolated from *Curcuma aeruginosa*, as well as compounds (imperatorin, **114**; phellopterin, **115**) isolated from *Angelica archangelica* (Figure 12), also shows antiviral properties against CV-B at IC_50_ values of 7.1, 15.6, and 3.9 μg/mL [52,124].

**Figure 13:**
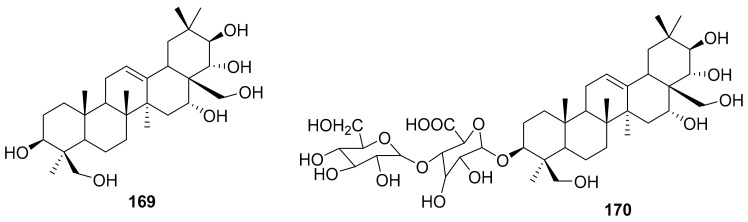
Isolated compounds from of *Aesculus turbinata*.

Kim et al. isolated ten oleanane-type triterpenoids from the seeds of *Aesculus turbinata* (Figure 13). The isolated compounds **169** and **170** showed potential antiviral properties against porcine epidemic diarrhea virus (PEDV). Western blotting showed that compounds **169** and **170** showed significant inhibition of nucleocapsid protein synthesis and inhibition of RNA expression of nucleocapsid and spike when treated in Vero cells at a concentration of 40 μM. Compound **169** was found to show inhibition of the RNA expression in a dose-dependent manner. Compound **169** was the more potent of the isolates; thus, further docking modeling of ARS-CoV 3CLpro (PDB ID code 3V3M) was performed. The docking study resulted in a proposed mechanism of action for **169** as a 3C-Chymotrypsin-Like protease (3CL protease) inhibitor [168].

**Figure 14:**
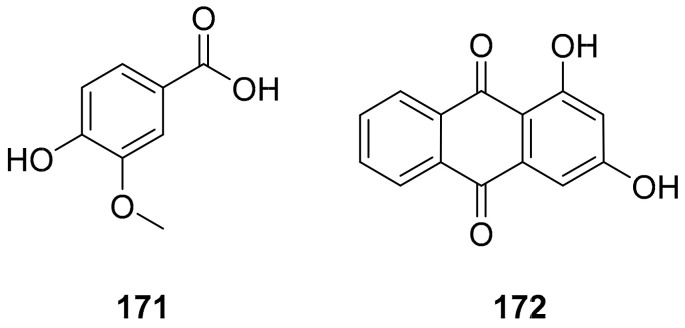
Isolated compounds from *Rubia cordifolia*.

Sun et al. used the aqueous extract from the aerial parts of *Rubia cordifolia* (RCAP) to explore its antiviral activity against the human rotavirus. Antiviral assays, qPCR, and other techniques were utilized to determine the potency of this extract. At concentrations of 15.63 mg/mL and above, the rotavirus becomes undetectable and accelerates rotavirus-induced apoptosis. The researcher also isolated compounds **171** and **172** (Figure 14) from RCAP; while no formal tests were done, previous research suggests that they may exhibit similar anti-rotavirus activity [169].

**Figure 15:**
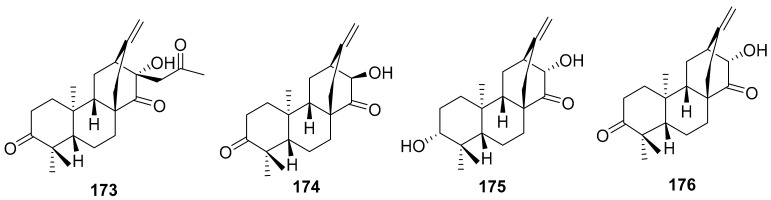
Isolated compounds from *Euphorbia ebracteolate*.

Human rhinovirus (HRV) is one of the most important causative etiological agents of the common cold. Even though upper respiratory infection because of HRV is mild and self-limiting, there are many reports that HRV infection leads to severe medical complications, including asthma aggravation. Wang et al. extracted unusual ent-atisane type diterpenoids with 2-oxopropyl skeleton (**173**–**176**) from the roots of *Euphorbia ebracteolate* (Figure 15) showing antiviral properties against human rhinovirus 3 (HRV3) with an IC_50_ value range from 25 to 90 μM [170].

Respiratory syncytial virus (RSV), an enveloped negative-sense RNA virus, is the most common cause of acute lower respiratory infections in infants and children. Every year, RSV causes millions of hospitalizations and thousands of deaths. Currently, very few drugs are available for the treatment of RSV, and new drug development against RSV is urgently needed. Plant sources are an attractive source for the identification of lead compounds or drug candidates for RSV. Isolated compounds from different plants that show potential antiviral properties against RSV are listed in Table 11.

Ebola virus (EBOV) is a negative-stranded RNA virus, which recently caused the outbreak in West Africa. The outbreak caused more than 28,000 cases and about 11,000 deaths. This epidemic reveals the need of an effective drug candidate for EBOV due to its mutations counteracting innate immune system responses. The EBOV VP35 protein is essential for viral inhibition of IFN production, and the protein is considered as an effective viral target. Petrillo et al. investigated the ethanolic extract of *Asphodelus microcarpus* for EBOV. The studies indicate that the ethanolic extract significantly reverted the EBOV VP35 inhibition of the vRNA-induced IFN response at concentrations of 3–0.1 μg/mL [174].

**Figure 16:**
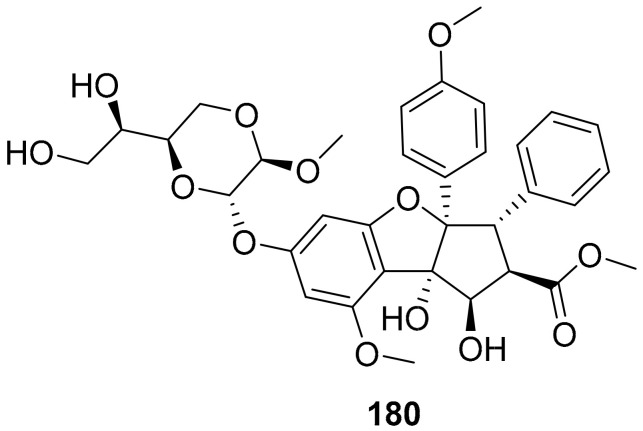
Structure of silvestrol (**180**).

Biedenkopf et al. identified silvestrol (**180**) (Figure 16) as a potential inhibitor (IC_50_: 10 nM) of EBOV replication. The authors isolated silvestrol (**180**) from the plant *Aglaia foveolate*. Effective silvestrol concentrations were non-toxic in the tested cell systems. Silvestrol could be considered as a potential drug candidate or lead molecule for EBOV [175].

In our search for plant extracts and isolated compounds for the antiviral properties against various viruses, we observed that polar extracts (aqueous or methanolic/ethanolic extracts) and polar secondary metabolites of the plant materials showed the most potency. We observed diverse classes of compounds isolated from different parts of various plants. We believe it is difficult to categorize based on chemical diversity. However, the predominant class of secondary metabolites reported for potential antiviral properties are polyphenolic compounds, glycosides, terpenoids, anthraquinones, and coumarins. In addition, most of the reported compounds show antiviral properties are oxygen-rich molecules, including antioxidants.

Many antioxidant molecules slow or stop viral virus replication and show antiviral properties [176]. The cellular injury due to viral infections caused by the over generation of free radicals has been linked to over 200 clinical disorders [177,178]. The overproduction of free radicals that lead to the development of oxidative stress is associated with pathogenic factors in a variety of viral infections [179].

In addition to the biological potential of the molecules, having balanced pharmacokinetic (ADME—Absorption, Distribution, Metabolism, and Excretion) properties of drug-like molecules is one the most difficult and challenging parts of the drug development process. We used a computational software STARDROP to determine the properties such as lipophilicity (logP), human intestine absorption (HIA), blood–brain barrier ability (BBB), hERG inhibition potential (hERG pIC_50_), rotatable bonds, hydrogen bond donor (HBD), hydrogen bond acceptor (HBA), and molecular weight (MW) [180]. The properties of the most effective natural compounds (included in this manuscript) isolated from different plants are shown in Table 12.

Among the potent compounds, most of them follow the “Rule of Five” with some violations. The hERG pIC_50_ values are also important to consider, since these values indicate possible cardiac toxicity (especially compounds with >5 hERG pIC_50_ value). We believe this compiled information, including the drug-like properties, will enrich the process of developing new potential antiviral drug candidates.

## 3. Methodology

The references considered for this review article were retrieved from PubMed, SciFinder, Springer, ScienceDirect, ACS, Google Scholar, and Wiley databases from 2015 to 2020, and the search keywords used antiviral combined with natural products and further filter by the plant(s). Both plant extracts and isolated compounds along with their IC_50_ and/or EC_50_ values were reported in this review. We also used the terms viruses, plants, H1N1, HIV, HSV, phytochemical, etc. to identify missing relevant articles to include in the review. The search strategy identified 1319 publications, and 102 references were excluded for duplication. We have also searched current clinical trials on natural products from plant source as potential therapy for viral infection using www.clinicaltrials.gov (accessed on 6 October 2021). Currently, there are 52 studies being conducted; however, most of the studies are preventive treatments (using heparin and vitamin C).

## 4. Conclusions

As viruses become more prevalent around the world, it is important to continue to look for new and improved antiviral drugs. Based on the extensive research efforts from 2015, there are a plethora of plant resources that show potential antiviral properties against various strains of the epidemic and pandemic-causing viruses. This review shows antiviral activity against the pandemic and epidemic-causing viruses: avian influenza A (H5N1 and H1N1), Ebola virus, and SARS-CoV-2. These viruses and others are responsible for the death of millions, warranting an expanded research effort into avenues that are not normally taken, such as natural resources. We have found that most polar components of the plants show antiviral properties. The secondary metabolites reported for antiviral properties are in the class of coumarins, polyphenolics, glycosides, and terpenoids. Modes of actions of the isolated compounds from plant sources may provide insight for the design of novel derivatives that show potent antiviral activity. Overall, this extensive review may serve as inspiration for the development of novel drug candidates that take advantage of the unique and diverse chemical structures of isolated compounds and extracts from plant sources, including those against drug-resistant and vaccine immunity escaping viral strains.

## Figures and Tables

**Table 1:** Active natural extracts against influenza strains.

S. No.	Plant Name	Plant Extract	Virus	Activity	Ref.
1	*Allium sativum*	Methanolic extract of roots	H1N1	EC_50_: 5 mg/mL	[32]
2	*Plumbago indica*	Ethanolic extract of roots	H1N1	EC_50_: 1 mg/mL	[32]
3	*Arachis hypogaea* L.	Peanut skin extracted with hexane	H1N1	IC_50_: 1.0–1.5 µg/mL	[33]
4	*Caesalpinia decapetala*	Aqueous ethanolic extract of leaves	H1N1	EC_50_: 5.7 µg/mL	[34]
5	*Carpesium abrotanoides* L.	Dried herbal ethanolic extraction	H1N1	IC_50_: 15.9 µM	[35]
			H3N2	IC_50_: 11.6 µM	
6	*Cayratia pedata*	DMSO extract of leaves	H1N1	IC_50_: 65.99 µg/mL	[36]
7	*Cayratia pedata*	DMSO extract of stem bark	H1N1	IC_50_: 20.50 µg/mL	[36]
8	*Diotacanthus albiflorus*	DMSO extract of leaves	H1N1	IC_50_: 60.09 µg/mL	[36]
9	*Diotacanthus albiflorus*	DMSO extract of stem bark	H1N1	IC_50_: 33.98 µg/mL	[36]
10	*Embelia Ribes*	Fruits extracted with ethyl acetate	H1N1	IC_50_: 0.2 µM	[37]
11	*Hippophae rhamnoides* L.	Methanolic extracts of leaves	H1N1	IC_50_: 7.2l µg/mL	[38]
12	*Hippophae rhamnoides* L.	Ethyl acetate extracts of leaves	H1N1	IC_50_: 10.3l µg/mL	[38]
13	*Murraya paniculata* L.	Petroleum ether extraction of plant leaves	H5N1	IC_50_: 0.15 µg/mL	[39]
14	*Piper longum*	Methanolic and chloroform extract from seeds	H1N1	IC_50_: 33.43–46.24 µg/mL	[40]
15	*Piper nigrum*	Methanolic and chloroform extract from seeds	H1N1	IC_50_: 17.47 µg/mL	[40]
16	*Polygonum chinense Linn*	Methanolic extract of dried and ground whole plant	H1N1	EC_50_: 38.4–55.5 µg/mL	[41]
17	*Poncirus trifoliata*	Seeds extracted with ethanol	H1N1	EC_50_: 2.51 µg/mL	[42]
18	*Psoralae Semen*	Aqueous extract of unknown part	H1N1	Inhibitory (%): 30	[43]
19	*Radix isatidis*	Hot methanol and ethanol extraction	H1N1	IC_50_: 3.34 mg/mL	[44]
20	*Ruta graveolens* L.	Petroleum ether extraction of plant leaves	H5N1	IC_50_: 7.8 µg/mL	[39]
21	*Strychnos minor*	DMSO extract of leaves	H1N1	IC_50_: 46.69 µg/mL	[36]
22	*Strychnos minor*	DMSO extract of stem bark	H1N1	IC_50_: 22.43 µg/mL	[36]
23	*Strychnos nux-vomica*	DMSO extract of leaves	H1N1	IC_50_: 33.36 µg/mL	[36]
24	*Strychnos nux-vomica*	DMSO extract of stem bark	H1N1	IC_50_: 23.60 µg/mL	[36]

Reference drugs: Ribavirin (EC_50_: 20.5–49.9 µM); Osehamivir (IC_50_: 0.015–0.025 µM); Oseltamivir (IC_50_: 3.71–6.44 µM).

**Table 2:** Isolated natural compounds against influenza strains.

S. No.	Plant Name (Part)	Compound	Virus	Activity	Ref.
1	*Forsythia suspensa* (Fruits)	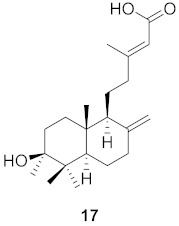	H1N1	IC_50_: 19.9 µM	[50]
2	*Forsythia suspensa* (Fruits)	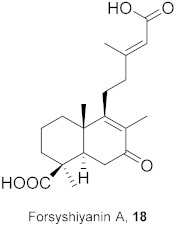	H1N1	IC_50_: 18.4 µM	[50]
3	*Forsythia suspensa* (Fruits)	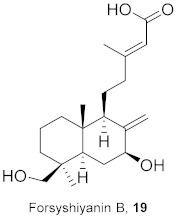	H1N1	IC_50_: 26.2 µM	[50]
4	*Forsythia suspensa* (Fruits)	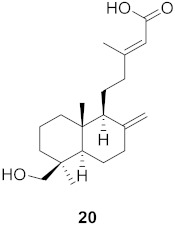	H1N1	IC_50_: 25.7 µM	[50]
5	*Forsythia suspensa* (Fruits)	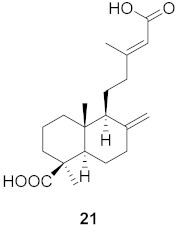	H1N1	IC_50_: 24.1 µM	[50]
6	*Forsythia suspensa* (Fruits)	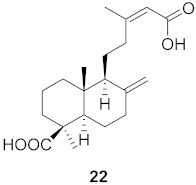	H1N1	IC_50_: 24.9 µM	[50]
7	*Forsythia suspensa* (Fruits)	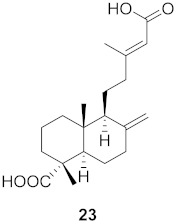	H1N1	IC_50_: 23.5 µM	[50]
8	*Forsythia suspensa* (Fruits)	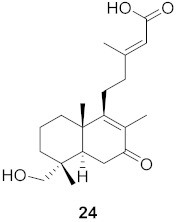	H1N1	IC_50_: 18.6 µM	[50]
9	*Basilicum polystachyon* (Whole plant)	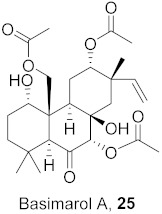	H1N1	IC_50_: 4.1 μM	[51]
			H3N3	IC_50_: 18 μM	
10	*Curcuma aeruginosa* (Rhizomes)	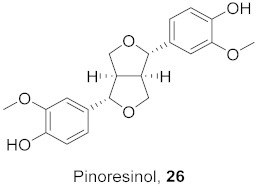	H1N1	IC_50_: 30.4 μg/mL	[52]
11	*Sonneratia paracaseolaris*	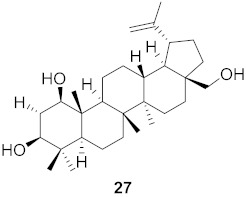	H1N1	IC_50_: 28.4 µg/mL	[53]
12	*Salvia plebeian R. Br* (Roots)	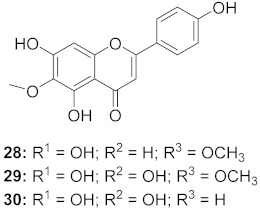	H1N1	IC_50_: 16.65–19.83 µM	[54]
13	*Ilex asprella* (Roots)	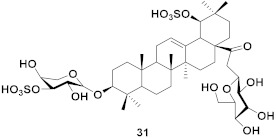	H1N1	EC_50_: 4.1 µM	[55]
14	*Ilex asprella*(Roots)	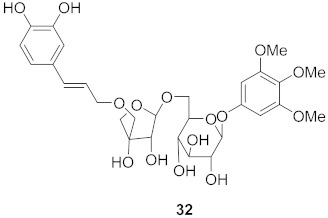	H1N1	EC_50_: 1.7 µM	[55]
15	*Abies beshanzuensis* (Bark)	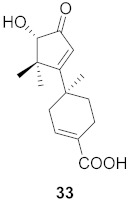	H3N2	IC_50_: 30.8 µg/mL	[56]
16	*Abies beshanzuensis* (Bark)	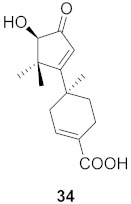	H3N2	IC_50_: 30.9 µg/mL	[56]
17	*Cleistocalyx operculatus* (Leaves)	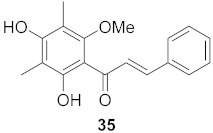	H1N1	IC_50_: 5.07 µM	[57]
			H9N2	IC_50_: 9.34 µM	
18	*Cleistocalyx operculatus* (Leaves)	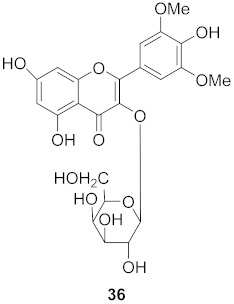	H1N1	IC_50_: 5.07 µM	[57]
			H9N2	IC_50_: 9.34 µM	
19	*Elaeocarpus tonkinesis* (Leaves and Twigs)	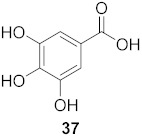	H1N1	EC_50_: 8.1 µg/mL	[58]
20	*Elaeocarpus tonkinesis* (Leaves and Twigs)	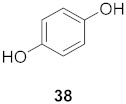	H1N1	EC_50_: 19.7 µg/mL	[58]

Reference drugs: Ribavirin (IC_50_: 24.6 µg/mL, EC50: 52.2–56.9 µM); Oseltamivir (IC_50_: 0.10 µM, EC50: 0.01–2.36 µM).

**Table 3:** Extracts from plants that are active against HIV.

S. No.	Plant name	Plant Extract	Virus	Activity	Ref.
1	*Artemisia campestris*	Aqueous ethanolic extract of the whole plant	HIV-1	IC_50_: 14.62 μg/mL	[62]
2	*Cassia Siberiana*	Chloroform methanolic extract of roots	HIV-1	IC_50_: 84.8 μg/mL	[63]
3	*Croton megalobotrys*	Chloroform methanolic extract of bark	HIV-1	IC_50_: 0.05 μg/mL	[63]
4	*Daphne gnidium* L.	Ethyl acetate extraction of branches	HIV-1	EC_50_: 0.08 μg/mL	[64]
5	*Eclipta alba*	Leaves extracted with chloroform	HIV-1	IC_50_: 250 μg/mL	[65]
6	*Euphorbia kansui*	Methanolic extract of roots	HIV-1	EC_50_: 110 ng/mL	[66]
7	*Terminalia chebula*	Methanolic/aqueous extract of fruit	HIV-1	IC_50_: ≤5 μg/mL	[67]
8	*Vitex doniana*	Chloroform methanolic extract of roots	HIV-1	IC_50_: 25 μg/mL	[63]

Reference drugs: Efavirenz (EC_50_: 0.0007–0.002 μM); Zidovudine (EC_50_: 0.005 μg/mL, 0.02 μM).

**Table 4:** Compounds isolated from plants against HIV.

S. No.	Plant Name (Part)	Compound	Virus	Activity	Ref.
1	*Clausena anisum-olens* (Leaves and twigs)	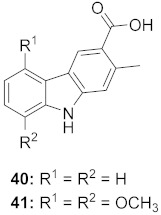	HIV	**40**: EC_50_: 2.4 μg/mL **41**: EC_50_: 3.7 μg/mL	[69]
2	*Manilkara zapota* (Fruit)	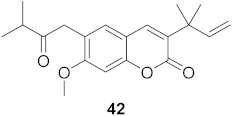	HIV	EC_50_: 8.69 μM	[70]
3	*Manilkara zapota* (Fruit)	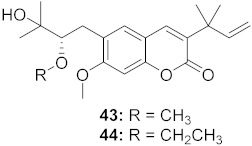	HIV	**43**: EC_50_: 0.33 μM **44**: EC_50_: 0.42 μM	[70]
4	*Manilkara zapota* (Fruit)	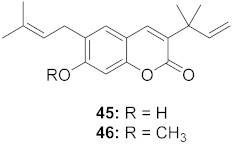	HIV	**45**: EC_50_: 2.28 μM **46**: EC_50_: 3.49 μM	[70]
5	*Manilkara zapota* (Fruit)	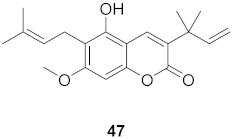	HIV	EC_50_: 4.26 μM	[70]
6	*Manilkara zapota* (Fruit)	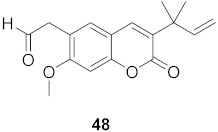	HIV	EC_50_: 0.97 μM	[70]
7	*Manilkara zapota* (Fruit)	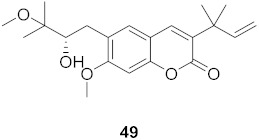	HIV	EC_50_: 5.26 μM	[70]
8	*Manilkara zapota* (Fruit)	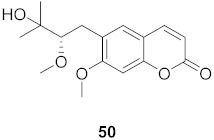	HIV	EC_50_: 6.73 μM	[70]
9	*Manilkara zapota* (Fruit)	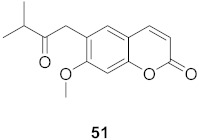	HIV	EC_50_: 0.12 μM	[70]
10	*Euphorbia semiperfoliata* (Whole plant)	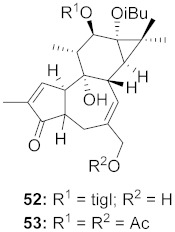	HIV-1	**52**: EC_50_: 0.013 μM **53**: EC_50_: 0.054 μM	[71]
11	*Salvia miltiorrhiza Bunge*	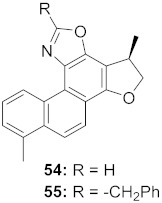	HIV-1	**54**: IC_50_: 0.03 μM **55**: IC_50_: 1.2 μM	[72]
12	*Marcetia taxifolia* (Aerial parts)	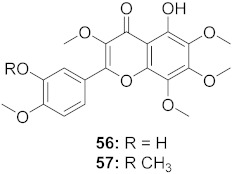	HIV-1	**56**: IC_50_: 4.1 μM **57**: IC_50_: 0.4 μM	[73]
13	*Justicia gendarussa* (Stem and bark)	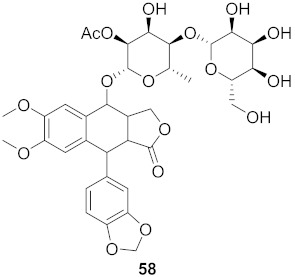	HIV-1	IC_50_: 15–21 nM	[74]
14	*Justicia gendarussa* (Root and stem)	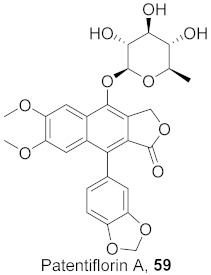	HIV- 1	IC_50_: 26.9 nM	[75]
15	*Rheum palmatum* L. and *Rheum officinale Baill* (Roots)	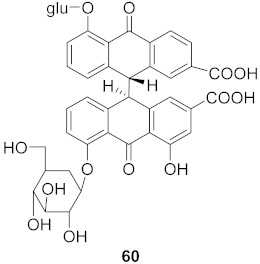	HIV	IC_50_: 1.9 μM	[76]
16	*Rheum palmatum* L. and *Rheum officinale Baill* (Roots)	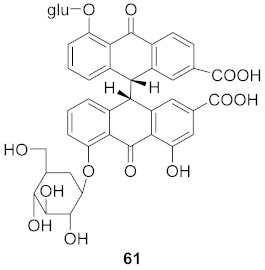	HIV	IC_50_: 2.1 μM	[76]
17	*Flueggea virosa* (Roots)	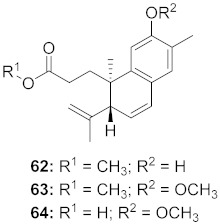	HIV	**62**: EC_50_: 19.2 μM **63**: EC_50_: 20.5 μM **64**: EC_50_: 40.1 μM	[77]
18	*Flueggea virosa* (Roots)	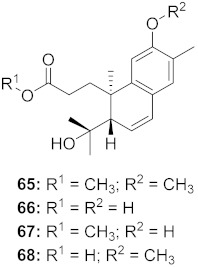	HIV	**65**: EC_50_: 51.8 μM **66**: EC_50_: >100 μM **67**: EC_50_: 87.8 μM **68**: EC_50_: 7.1 μM	[77]
19	*Flueggea virosa* (Roots)	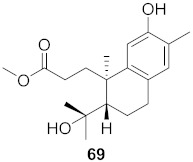	HIV	EC_50_: 58.0 μM	[77]
20	*Flueggea virosa* (Roots)	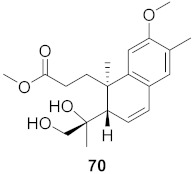	HIV	EC_50_: >100 μM	[77]
21	*Flueggea virosa* (Roots)	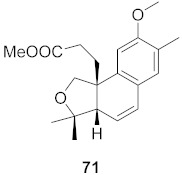	HIV	EC_50_: 53.9 μM	[77]
22	*Flueggea virosa* (Roots)	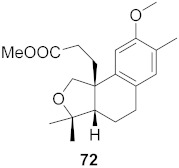	HIV	EC_50_: 48.6 μM	[77]
23	*Flueggea virosa* (Roots)	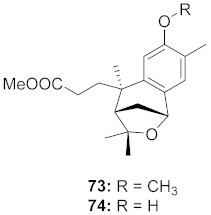	HIV	**73**: EC_50_: 40.6 μM **74**: EC_50_: >100 μM	[77]
24	*Flueggea virosa* (Roots)	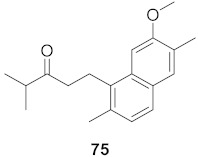	HIV	EC_50_: 69.4 μM	[77]
25	*Chloranthus japonicus* (Roots)	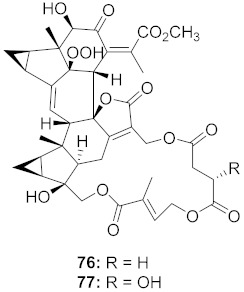	HIV-1	**76**: EC_50_: 3.08 μM **77**: EC_50_: 3.29 μM	[78]
26	*Chloranthus japonicus* (Roots)	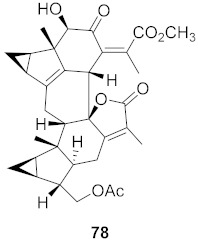	HIV-1	EC_50_: 5.41 μM	[78]
27	*Kaempferia pulchra* (Rhizomes)	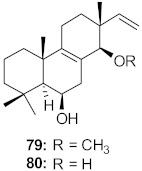	HIV-1	IC_50_: 1.56–6.25 μM	[79]
28	*Kaempferia pulchra* (Rhizomes)	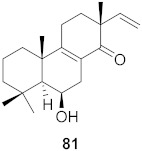	HIV-1	IC_50_: 1.56–6.25 μM	[79]
29	*Kaempferia pulchra* (Rhizomes)	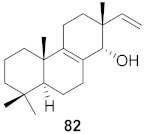	HIV-1	IC_50_: 1.56–6.25 μM	[79]
30	*Kaempferia pulchra* (Rhizomes)	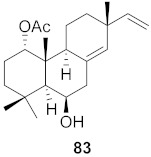	HIV-1	IC_50_: 1.56–6.25 μM	[79]
31	*Kaempferia pulchra* (Rhizomes)	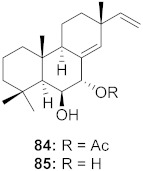	HIV-1	IC_50_: 1.56–6.25 μM	[79]
32	*Stillingia lineata* (Bark)	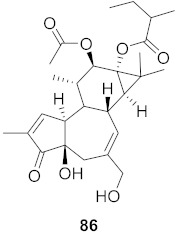	HIV-1	EC_50_: 0.271 μM	[80]
			HIV-2	EC_50_: 0.107 μM	
33	*Stillingia lineata* (Bark)	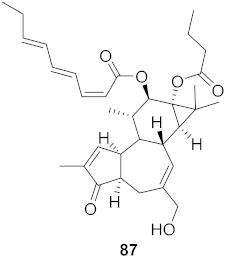	HIV-1	EC_50_: 0.233 μM	[80]
			HIV-2	EC_50_: 0.174 μM	
34	*St35illingia lineata* (Bark)	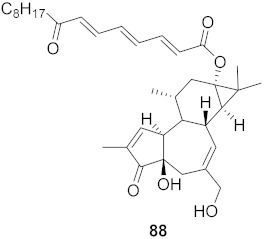	HIV-1	EC_50_: 0.043 μM	[80]
			HIV-2	EC_50_: 0.018 μM	
35	*Moquiniastrm floribundum* (Leaves)	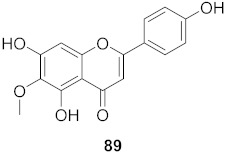	HIV-1	IC_50_: 0.345 mM	[81]
36	*Moquiniastrm floribundum* (Leaves)	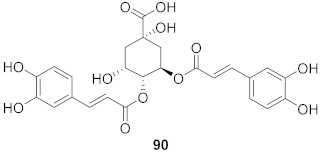	HIV-1	IC_50_: 0.240 mM	[81]
37	*Moquiniastrm floribundum* (Leaves)	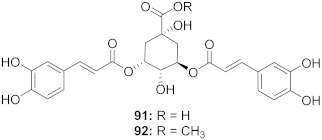	HIV-1	**91**: IC_50_: 0.315 mM **92**: IC_50_: 0.250 mM	[81]
38	*Moquiniastrm floribundum* (Leaves)	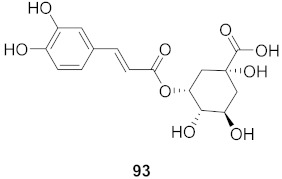	HIV-1	IC_50_: 0.374 mM	[81]
39	*Moquiniastrm floribundum* (Leaves)	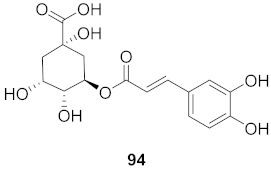	HIV-1	IC_50_: 0.489 mM	[81]

Reference drugs: Zidovudine (EC_50_: 0.005 μg/mL, 0.02 μM); Prostratin (EC_50_: 0.226 μM); Efavirenz (IC_50_: 0.0007–0.002 μM); Nevaripine (IC_50_: 0.1–0.5 μM); Honokiol (IC_50_: 45.9 μM); Myricetin (0.2 mM); Foscarnet (0.001 mM).

**Table 5:** Plant extracts with antiviral activity against arthropod-borne flaviviruses.

S. No.	Plant Name	Plant Extract	Virus	Activity	Ref.
1	*Andrographis paniculata*	Pure andrographolide in DMSO	DENV2	EC_50_: 21.304 µM	[93]
2	*Dryopteris crassirhizoma*	Aqueous extract of whole plant	DENV	IC_50_: 130 µg/mL	[94]
3	*Euphorbia amygdaloides semiperfoliata*	Ethyl acetate extract of whole plant	CHIKV	EC_50_: <0.8 µg/mL	[95]
4	*Euphorbia characias*	Ethyl acetate extract of stems	CHIKV	EC_50_: 2.9 µg/mL	[95]
5	*Euphorbia hyberna insularis*	Ethyl acetate extract of aerial parts	CHIKV	EC_50_: 1.0 µg/mL	[95]
6	*Euphorbia pithyusa*	Ethyl acetate extract of leaves	CHIKV	EC_50_: <0.8 µg/mL	[95]
7	*Euphorbia pithyusa*	Ethyl acetate extract of stems	CHIKV	EC_50_: <0.8 µg/mL	[95]
8	*Euphorbia pithyusa*	Methanolic and ethyl acetate extract of roots.	CHIKV	EC_50_: <0.8 µg/mL	[95]
9	*Euphorbia segetalis pinea*	Ethyl acetate extract of roots	CHIKV	EC_50_: 1.8 µg/mL	[95]
10	*Euphorbia segetalis pinea*	Ethyl acetate extract of arial parts	CHIKV	EC_50_: 3.7 µg/mL	[95]
11	*Euphorbia segetalis pinea*	Ethyl acetate extract of stems	CHIKV	EC_50_: 3.5 µg/mL	[95]
12	*Euphorbia spinosa*	Ethyl acetate extract of roots	CHIKV	EC_50_: <0.8 µg/mL	[95]
13	*Euphorbia spinosa*	Methanolic extract of roots	CHIKV	EC_50_: 2.3 µg/mL	[95]
14	*Euphorbia spinosa*	Ethyl acetate extract of stems	CHIKV	EC_50_: 3.4 µg/mL	[95]
15	*Justicia adhatoda*	Aqueous extract of leaf	DENV	IC_50_: 60 µg/mL	[96]
16	*Morus alba*	Aqueous extract of the whole plant	DENV	IC_50_: 221 µg/mL	[94]
17	*Psidium guajoava*	Aqueous extract of leaf	DENV	IC_50_: 60 µg/mL	[96]
18	*Syzygium campanulatum*	Ethyl acetate extract of leaves	DENV2	Inhibitory (%): 64.77	[97]
19	*Syzygium grande*	Ethyl acetate extract of leaves	DENV2	Inhibition (%): 61.46	[97]

**Table 6:** Compounds isolated from plants that are active against arthropod-borne flaviviruses.

S. No.	Plant Name (Part)	Compound	Virus	Activity	Ref
1	*Basilicum poly-stachyon*	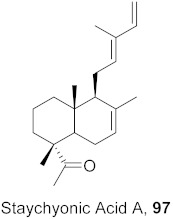	DENV	IC_50_: 1.4 µM	[99]
2	*Basilicum polystachyon* (Whole plant)	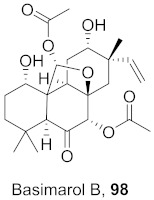	WNV	IC_50_: 100 μM	[51]
3	*Mammea americana* (Seeds)	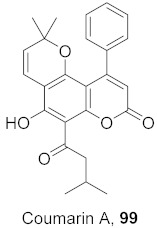	DENV2	EC_50_: 9.6 µg/mL	[100]
			CHIKV	EC_50_: 10.7 µg/mL	
4	*Mammea americana* (Seeds)	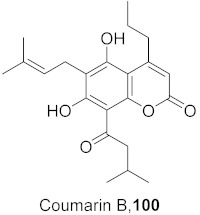	DENV2	EC_50_: 2.6 µg/mL	[100]
			CHIKV	EC_50_: 0.5 µg/mL	
5	*Diospyros Ebenaceae* (Bark)	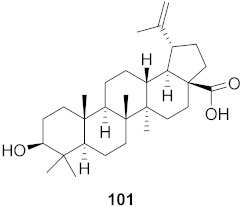	DENV	IC_50_: 6.6 µM	[101]
6	*Diospyros Ebenaceae* (Bark)	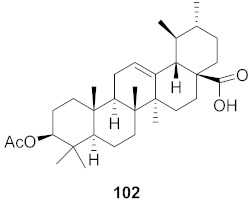	DENV	IC_50_: 7.0 µM	[101]
7	*Diospyros Ebenaceae* (Bark)	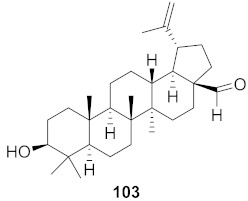	DENV	IC_50_: 6.1 µM	[101]
8	*Diospyros Ebenaceae* (Bark)	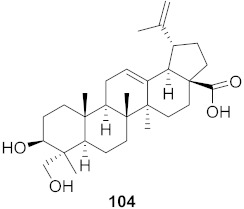	DENV	IC_50_: 5.3 µM	[101]
9	*Melia azedarach* (Fruits)	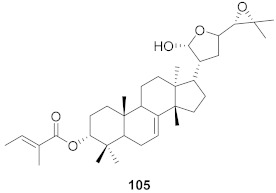	DENV2	EC_50_: 3.0 µM	[102]
10	*Melia azedarach* (Fruits)	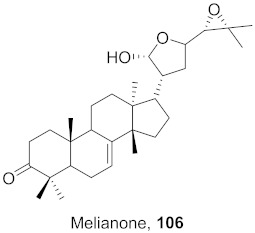	DENV2	EC_50_: 12 µM	[102]
11	*Stillingia lineata* (Bark)	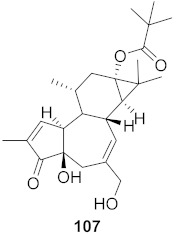	CHIKV	EC_50_: 1.2 μM	[80]
12	*Stillingia* *lineata* (Stem bark)	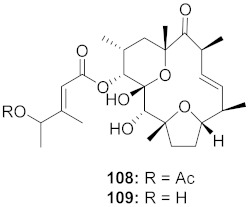	CHIKV	**108**: EC_50_: 7 μM **109**: EC_50_: 34 μM	[103]
13	*Stillingia lineata* (Bark)	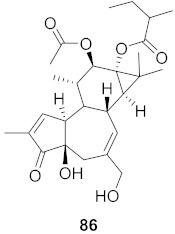	CHIKV	EC_50_: 3.3 μM	[80]
14	*Stillingia lineata* (Bark)	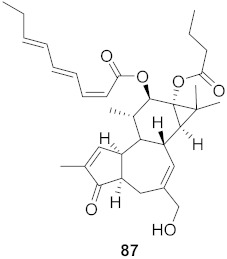	CHIKV	EC_50_: 1.4 μM	[80]
			SINV	EC_50_: 5.0 μM	
15	*Stillingia lineata* (Bark)	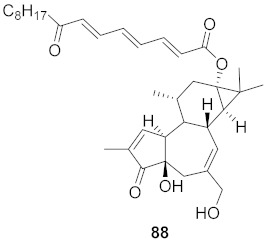	CHIKV	EC_50_: 2.2 μM	[80]
			SINV	EC_50_: 11 μM	
16	*Euphorbia semiperfoliata* (Whole plant)	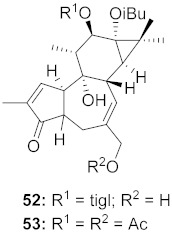	CHIKV	**52**: EC_50_: 1.0 μM **53**: EC_50_: 0.44 μM	[71]

**Table 7:** Extracts from plants active against HSV.

S. No.	Plant Name	Plant Extract	Virus	Activity	Ref.
1	*Arctium lappa* L.	Ethanolic extract of fruits	HSV-1	IC_50_: 400 μg/mL	[112]
2	*Epimedium koreanum*	Herbal aqueous extraction	HSV	EC_50_: 0.62 μg/mL	[113]
3	*Eucalyptus sideroxylon Cunn. ex Woolls*	Methanolic extract of leaves	HSV-2	IC_50_: 199.34 μg/mL	[114]
4	*Juncus Compressus*	Whole plant extracted with methanol	HSV-2	IC_50_: 12.4 μM	[115]
5	*Punica granatum* L.	Aqueous extract of pomegranate rind	HSV	EC_50_: 0.02 μg/mL	[116]
6	*Ribes multiflorum*	Methanol and aqueous extraction of leaves and fruit	HSV-1	EC_50_: 9710 μg/mL	[117]
7	*Ribes uva-crispa*	Methanol and aqueous extraction of leaves and fruit	HSV-1	EC_50_: 9710 μg/mL	[117]
8	*Rosmarinus officinalis*	Aqueous extract of the whole plant	HSV-1	EC_50_: 67.34 μg/mL	[118]
9	*Syzygium jambos*	Ethanolic extract of leaves	HSV-1	IC_50_: 50.00 μg/mL	[119]
10	*Terminalia chebula Retz*	Ethanolic extract of fruit	HSV-2	IC_50_: 0.01 μg/mL	[120]

Reference drug: Acyclovir (EC_50_: 0.8–2.1 μg/mL, 0.41 μM; IC_50_: 0.1 μg/mL).

**Table 8:** Compounds isolated from plants against HSV.

S. No.	Plant Name (Part)	Compound	Virus	Activity	Ref.
1	*Houttuynia cordata*	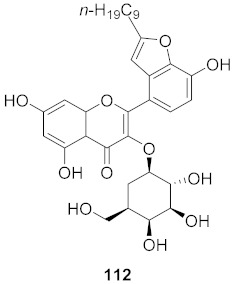	HSV	IC_50_: 23.5 μM	[122]
2	*Boswellia serrata* (Oleo-gum-resin)	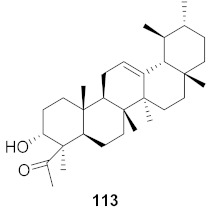	HSV-1	EC_50_: 5.2 μg/mL	[123]
3	*Angelica archangelica* L. (Fruits)	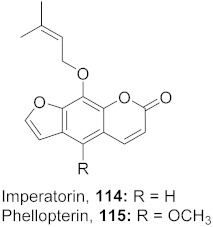	HSV-1	**114**: IC_50_: 15.62 μg/mL **115**: IC_50_: 3.90 μg/mL	[124]
4	*Rhododendron capitatum* (Aerial parts)	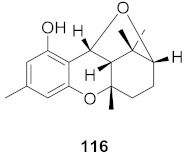	HSV-1	IC_50_: 4.2 μM	[125]
5	*Cnidium monnieri* (Fruit)	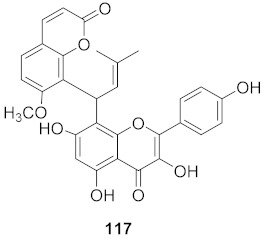	HSV-1	IC_50_: 1.23 μM	[126]
6	*Kalanchoe daigremontiana* (Leaves)	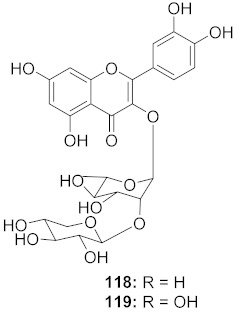	HSV-1	EC_50_: 0.97 μg/mL	[127]
			HSV-2	EC_50_: 0.72 μg/mL	
7	*Morus alba* L.	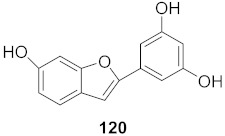	HSV-1	IC_50_: 2.2 ± 0.1 μg/mL	[128]
			HSV-2	IC_50_: 2.5 ± 0.3 μg/mL	
8	*Morus alba* L.	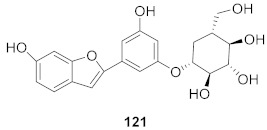	HSV-1	IC_50_: 5.0 μg/mL	[128]
			HSV-2	IC_50_: 3.2 μg/mL	
9	*Morus alba* L.	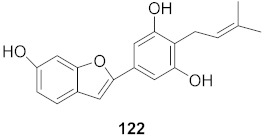	HSV-1	IC_50_: 8.4 μg/mL	[128]
			HSV-2	IC_50_: 8.2 ± 0.4 μg/mL	
10	*Morus alba* L.	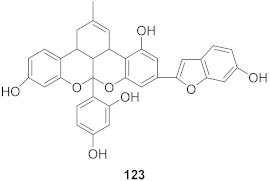	HSV-1	IC_50_: 5.2 μg/mL	[128]
			HSV-2	IC_50_: 3.7 μg/mL	
11	*Morus alba* L.	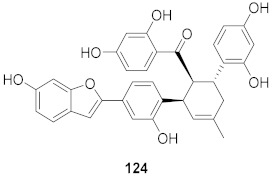	HSV-1	IC_50_: 12.5 μg/mL	[128]
			HSV-2	IC_50_: 12.5 μg/mL	
12	*Morus alba* L.	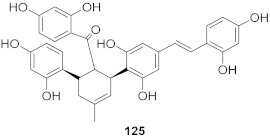	HSV-1	IC_50_: 6.3 μg/mL	[128]
			HSV-2	IC_50_: 25.0 μg/mL	
13	*Camellia sinensis* (Leaves)	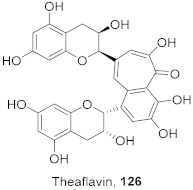	HSV-1	EC_50_: 50 μM	[129]
14	*Camellia sinensis* (Leaves)	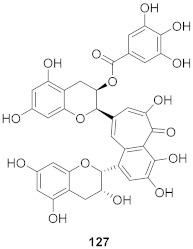	HSV-1	EC_50_: 25 μM	[129]
15	*Camellia sinensis* (Leaves)	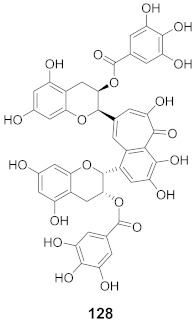	HSV-1	EC_50_: 20 μM	[129]
16	*Kalanchoe pinnata* (Root)	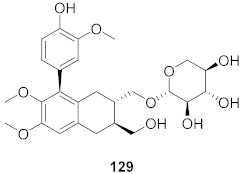	HSV-1	EC_50_: 0.97 μg/mL	[130]
			HSV-2	EC_50_: 0.72 μg/mL	
17	*Kalanchoe pinnata* (Root)	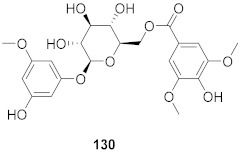	HSV-1	EC_50_: 0.97 μg/mL	[130]
			HSV-2	EC_50_: 0.72 μg/mL	

Reference drug: Acyclovir (EC_50_: 0.8–2.1 μg/mL, 0.41 μM; IC_50_: 0.1 μg/mL).

**Table 9:** Extracts from plants against hepatitis virus.

S. No.	Plant Name	Plant Extract	Virus	Activity	Ref.
1	*Abutilon figarianum*	Ethanolic then dichloromethane extract of whole plant	HBV	IC_50_: 99.76 μg/mL	[134]
2	*Acacia oerfota*	Total ethanolic extract of whole plant	HBV	IC_50_: 101.46 μg/mL	[134]
3	*Alectryon serratus*	Ethanolic extract of leaves	HCV	IC_50_: 9.8 μg/mL	[135]
4	*Alectryon serratus*	Chloroform methanolic extract of leaves	HCV	IC_50_: 1.2 μg/mL	[135]
5	*Alectryon serratus*	Chloroform methanolic and water extract of leaves	HCV	IC_50_: 0.43 μg/mL	[135]
6	*Boerhavia diffusa*	Methanolic extract of whole plant	HCV	IC_50_: 12.5–25 μM	[136]
7	*Capparis decidua*	Ethanolic then aqueous extract of the whole plant	HBV	IC_50_: 66.82 μg/mL	[134]
8	*Coccinea grandis*	Total ethanolic extract of whole plant	HBV	IC_50_: 31.57 μg/mL	[134]
9	*Corallocarpus epigeus*	Total ethanolic extract of whole plant	HBV	IC_50_: 71.9 μg/mL	[134]
10	*Curcuma domestica*	Dried powder of rhizomes was extracted with ethanol	HCV	IC_50_:1.68 μg/mL	[137]
11	*Curcuma heyneana*	Dried powder of rhizomes was extracted with ethanol	HCV	IC_50_: 5.49 μg/mL	[137]
12	*Curcuma xanthorrhiza*	Dried powder of rhizomes was extracted with ethanol	HCV	IC_50_:4.93 μg/mL	[137]
13	*Fumaria parviflora*	Ethanolic then hexane extract of the whole plant	HBV	IC_50_: 35.44 μg/mL	[134]
14	*Glycine max*	A fermented extract of defatted soybean meal	HAV	IC_50_: 27 μg/mL	[138]
15	*Glycine max*	A fermented extract of defatted soybean meal with Aspergillus fumigatus F-993	HAV	IC_50_: 8.60 μg/mL	[138]
16	*Glycine max*	A fermented extract of defatted soybean meal with A. awamori FB-113	HAV	IC_50_: 16.88 μg/mL	[138]
17	*Guiera senegalensis*	Ethanolic then dichloromethane extract of the whole plant	HBV	IC_50_: 10.65 μg/mL	[134]
18	*Indigofera caerulea*	Methanolic extract of whole plant	HBV	IC_50_: 73.21 μg/mL	[134]
19	*Juncus maritimus Lam.*	Methanolic extract of rhizomes	HCV	Inhibition (%): >50	[139]
20	*Lentinula edodes*	Hot water extraction of mycelia	HCV	IC_50_: 5 μg/mL	[140]
21	*Limonium sinense*	Aqueous extract from underground part of plant	HCV	EC_50_: 9.71 μg/mL CC_50_: 343.47 μg/mL	[141]
22	*Phyllanthus reticulates* Poir.	Aqueous and ethanol extracts	HBV	EC_50_: 0.56μg/mL	[142]
23	*Pinus pinaster*	Pine extract from bark	HCV	IC_50_: 5.78 μg/mL EC_50_: 4.33 μg/mL	[143]
24	*Pulicaria crispa*	Ethyl acetate extract of whole plant	HBV	IC_50_: 14.45 μg/mL	[134]
25	*Taraxacum officinale*	Methanolic extract of leaves	HCV	Inhibition (%): >65%	[144]
26	*Valeriana wallichii*	Methanolic extract of roots	HCV	CC_50_: 252.2 μg/mL	[145]

**Table 10:** Compounds isolated from plants with antiviral activity against hepatitis virus.

S. No.	Plant Name (Part)	Compound	Virus	Activity	Ref.
1	*Phyllantus acidus* (Stem)	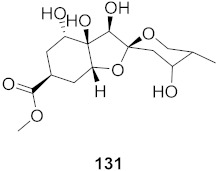	HBV	IC_50_: 11.2 μM	[146]
2	*Phyllantus acidus* (Stem)	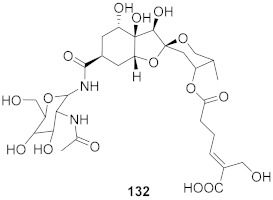	HBV	IC_50_: 57.1 μM	[146]
3	*Vitis vinifera* (Root)	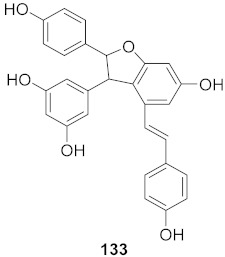	HCV	EC_50_: 0.006 μM	[147]
4	*Vitis vinifera* (Root)	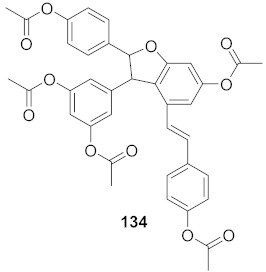	HCV	EC_50_: 2.37 μM	[147]
5	*Wikstroemia chamaedaphne* (Buds)	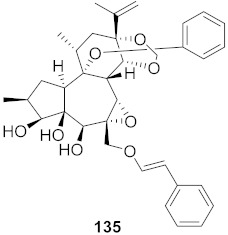	HBV	IC_50_: 46.5 μg/mL	[148]
6	*Wikstroemia chamaedaphne* (Buds)	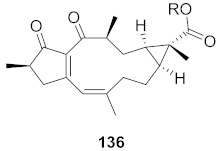	HBV	IC_50_: 88.3 μg/mL	[148]
7	Multiple *Fumaria* and *Corydalis* species from Turkey	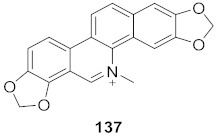	HBV	IC_50_: 15 mg	[149]
8	Multiple *Fumaria* and *Corydalis* species from Turkey	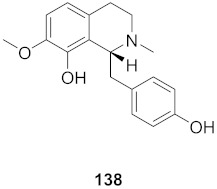	HBV	IC_50_: 23 mg	[149]
9	*Candida albicans* (Root)	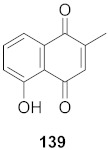	HCV	IC_50_: 0.57 μM/L	[150]
10	*Cyanara Cardunculus L.var. sylvestris (Lam.) Fiori* (Leaves)	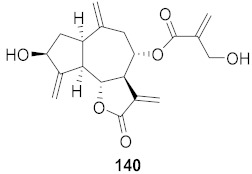	HCV	EC_50_: 0.4–1.4 μM	[151]
11	*Cyanara Cardunculus L.var. sylvestris (Lam.) Fiori* (Leaves)	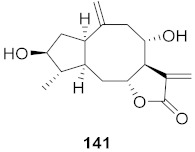	HCV	EC_50_: 2.7–14.0 μM	[151]
12	Green Tea	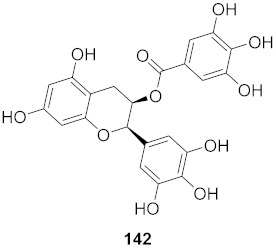	HBV	CC_50_: 247.28 μM	[152]
13	*Caulis trachelospermi*	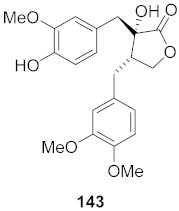	HCV	IC_50_: 0.325 μg/mL	[101]
14	*Selaginella moellendorffii*	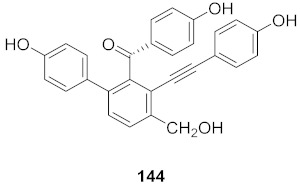	HBV	IC_50_: 0.026 μg/mL	[153]
15	*Phyllanthus urinaria*	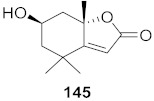	HCV	EC_50_: 2.48 μM	[154]
16	*Viola diffusa* Ging (Whole plant)	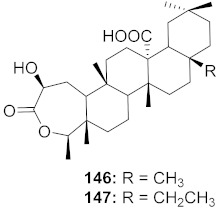	HBV	**146**: IC_50_: 26.2 μM **147**: IC_50_: 33.7 μM	[155]
17	*Viola diffusa* Ging (Whole plant)	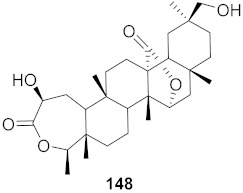	HBV	IC_50_: 104.0 μM	[155]
18	*Viola diffusa* Ging (Whole plant)	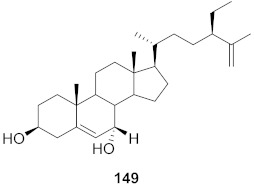	HBV	IC_50_: 62.0 μM	[155]
19	*Viola diffusa* Ging (Whole plant)	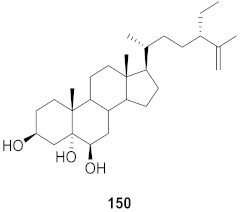	HBV	IC_50_: 32.7 μM	[155]
20	*Viola diffusa* Ging (Whole plant)	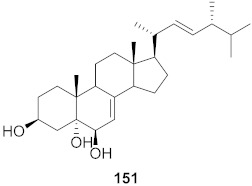	HBV	IC_50_: 112.8 μM	[155]
21	*Perovskia atriplicifolia* (Whole plant)	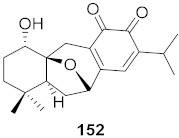	HBV	IC_50_: 1.03 μM	[156]
22	*Perovskia atriplicifolia* (Whole plant)	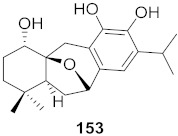	HBV	IC_50_: 0.59 μM	[156]
23	*Maytrenus ilicifolia* (Root bark)	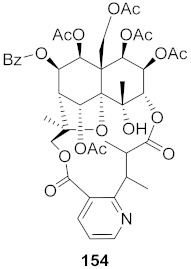	HCV	EC_50_: 2.3 μM	[157]
24	*Peperomia blanda* (Aerial parts)	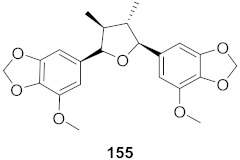	HCV	EC_50_: 4.0 μM	[157]
25	*Peperomia blanda* (Aerial parts)	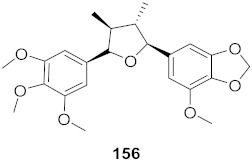	HCV	EC_50_: 8.2 μM	[157]
26	*Peperomia blanda* (Aerial parts)	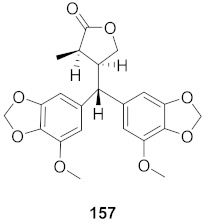	HCV	EC_50_: 38.9 μM	[157]
27	*Illicium jiadifengpi* (Fruits)	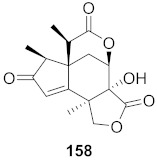	HBV	Inhibitory (%): 28.85	[158]
28	*Illicium jiadifengpi* (Fruits)	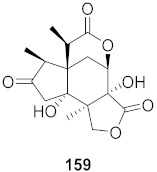	HBV	Inhibitory (%): 37.93	[158]
29	*Chloranthus japonicus* (Roots)	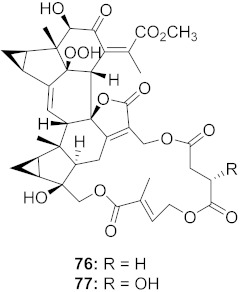	HCV	**76**: EC_50_: 3.07 μM **77**: EC_50_: 9.34 μM	[78]
30	*Chloranthus japonicus* (Roots)	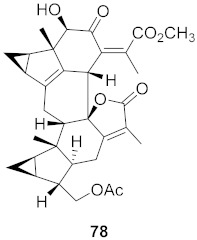	HCV	EC_50_: 1.62 μM	[78]
31	*Aloe vera* (Leaves)	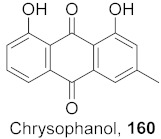	HBV	Inhibitory (%): 62	[159]
32	*Aloe vera* (Leaves)	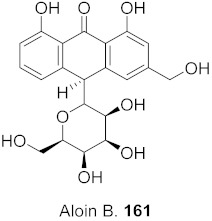	HBV	Inhibitory (%): 61	[159]
33	*Aloe vera* (Leaves)	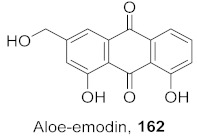	HBV	Inhibitory (%): 83	[159]

Reference drug: Lamivudine (IC_50_: 23.50 mM); Plumbagin (IC_50_: 0.57 µM).

**Table 11:** Compounds isolated from plants with antiviral activity against RSV.

S. No.	Plant Name (Part)	Compound	Virus	IC_50_/EC_50_	Ref.
1	*Lilium speciosum var gloriosoides Barker* (Bulbs)	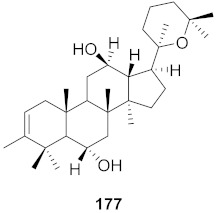	RSV	IC_50_: 2.9 μg/mL	[171]
2	*Lilium speciosum var gloriosoides Barker* (Bulbs)	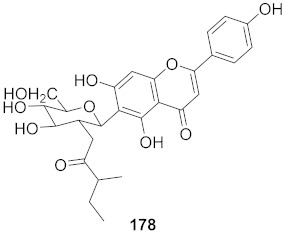	RSV	IC_50_: 2.1 μg/mL	[172]
3	*Erycibe obtusifolia*	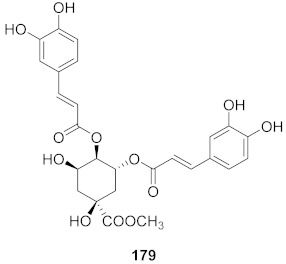	RSV A2	EC_50_: 0.52 μg/mL	[173]
			RSV Long	EC_50_: 0.59 μg/mL	

Reference drug: Ribavirin (EC_50_: 2.42–2.63 μg/mL).

**Table 12:** Drug-likeness properties of selected potential natural products.

Entry	Compound	logP	HIA	BBB	hERG pIC_50_	Rotatable Bonds	HBD	HBA	MW
1	**25**	1.991	+	-	3.836	8	2	9	494.6
2	**31**	1.129	-	-	4.580	10	8	18	925.1
3	**32**	−0.989	-	-	4.561	13	7	15	626.6
4	**43**	3.258	+	-	5.332	7	1	5	360.4
5	**44**	3.635	+	-	5.456	8	1	5	374.5
6	**48**	2.752	+	+	4.855	5	0	4	286.3
7	**51**	2.307	+	+	4.953	4	0	4	260.3
8	**54**	5.480	+	+	6.054	0	0	3	289.3
9	**55**	7.230	+	+	6.668	2	0	3	379.5
10	**56**	2.076	-	-	4.810	6	2	9	404.4
11	**57**	2.270	-	-	4.872	7	1	9	418.4
12	**86**	3.411	+	-	4.463	7	2	7	474.6
13	**88**	4.945	+	-	5.576	14	2	6	578.8
14	**97**	5.158	+	+	5.625	4	0	1	300.5
15	**100**	4.480	+	-	5.680	7	2	5	372.5
16	**105**	5.770	+	-	5.498	5	1	5	554.8
17	**107**	3.564	+	-	4.708	4	2	5	416.6
18	**117**	4.049	+	-	5.981	5	4	9	528.5
19	**118**	−0.320	-	-	4.146	5	8	14	564.5
20	**119**	−0.504	-	-	3.960	5	9	15	580.5
21	**129**	0.348	-	-	4.587	8	5	10	506.5
22	**130**	−0.473	-	-	4.216	9	5	12	482.4
23	**133**	3.561	+	-	5.617	4	5	6	454.5
24	**139**	2.345	+	+	3.865	0	1	3	188.2
25	**140**	1.284	+	-	3.963	4	2	6	346.4
26	**143**	2.531	+	-	4.917	7	2	7	388.4
27	**144**	3.605	+	-	5.396	6	4	5	436.5
28	**153**	3.472	+	-	5.208	1	3	4	332.4
29	**179**	1.414	-	-	4.310	10	6	12	530.5
30	**180**	0.821	+	-	4.432	11	4	13	654.7
31	Ribavirin	−1.85			3.325	3	4	9	244.2
32	Oseltamivir	1.767	+	-	3.737	9	2	6	312.4
33	Efavirenz	4.013	+	+	4.992	3	1	3	315.7
34	Zidovudine	−0.018	+	-	4.089	3	2	9	267.2
35	Prostratin	1.971	+	-	4.365	3	3	6	390.5
36	Nevaripine	1.828	+	-	4.890	1	0	5	300.7
37	Honokiol	4.362	+	+	5.358	5	2	2	266.3
38	Myricetin	1.303	-	-	4.274	1	6	8	318.2
39	Foscarnet	−1.535	+	-	2.667	1	3	5	126.0
40	Acyclovir	−1.649	-	-	4.302	4	3	8	225.2
41	Lamivudine	−1.036	+	-	3.955	2	2	6	229.3
42	Plumbagin	2.345	+	+	3.865	0	1	3	188.2

logP: lipophilicity; HIA: human intestine absorption (+ = can absorb through intestine, - = cannot absorb through intestine); BBB: blood–brain barrier (+ = can cross BBB, - = cannot cross BBB); hERG pIC_50_: hERG (human ether-a-go-go-related gene) activity (pIC_50_); HBD: hydrogen bond donor; HBA: hydrogen bond acceptor; MW: molecular weight.

## Abbreviations

229E	Human coronavirus
AV	Adenovirus
B/Lee/40	Influenza B/Lee/40
CHIKV	Chikungunya virus
CV-B	Coxsackievirus B
CV	Coxsackieviruses
CyHV-3	Cyprinid herpesvirus 3
DENV	Dengue virus
EBV	Epstein–Barr virus
EHV-1	Equid herpesvirus 1
EV	Ebola virus
FV	Flavivirus
H1N1	Influenza virus
H3N2	Influenza A/Victoria virus
H5N1	Avian influenza virus
H9N2	Novel Reassortant avian influenza A virus
HAV	Hepatitis A Virus
HBV	Hepatitis B Virus
HCoV 229E	Human coronavirus
HCV	Hepatitis C virus
HIV-1	Human immunodeficiency virus 1
HIV-2	Human immunodeficiency virus 2
HIV	Human immunodeficiency virus
HR3V	Human rhinovirus 3 virus
HRoV	Human rotavirus
HSV	Herpes simplex viruses
HRV	Human rhino virus
HSV-1	Herpes simplex viruses 1
HSV-2	Herpes simplex viruses 2
HuNoVs	Human noroviruses
MV	Measles virus
MNV-1	Murine Norovirus-1
NDV	Newcastle disease virus
POV	Poliovirus
PRRSV	Porcine reproductive and respiratory syndrome virus
PRV	Pseudorabies Virus
PV	Pestivirus
PV1	Picornavirus
RSV	Respiratory syncytial virus
SARS-CoV-2	COVID19
SFV	Semliki forest virus
SINV	Sindbis virus
SuHV-1	Suid herpesvirus 1
TMV	Tobacco mosaic virus
VSV	Vesicular stomatitis virus
VV	Vaccinia virus
WNV	West Nile virus
WSSV	White spot syndrome virus
YFV	Yellow fever virus
ZIKV	Zika virus
